# A Dynamic Tandem Repeat in Monocotyledons Inferred from a Comparative Analysis of Chloroplast Genomes in Melanthiaceae

**DOI:** 10.3389/fpls.2017.00693

**Published:** 2017-05-22

**Authors:** Hoang Dang Khoa Do, Joo-Hwan Kim

**Affiliations:** Plant Systematics Laboratory, Department of Biological Science, Gachon UniversitySeongnam, South Korea

**Keywords:** *Xerophyllum tenax*, *Heloniopsis tubiflora*, chloroplast genome, tandem repeats, slipped-strand mispairing, Parideae, Melanthiaceae, Liliales

## Abstract

Chloroplast genomes (cpDNA) are highly valuable resources for evolutionary studies of angiosperms, since they are highly conserved, are small in size, and play critical roles in plants. Slipped-strand mispairing (SSM) was assumed to be a mechanism for generating repeat units in cpDNA. However, research on the employment of different small repeated sequences through SSM events, which may induce the accumulation of distinct types of repeats within the same region in cpDNA, has not been documented. Here, we sequenced two chloroplast genomes from the endemic species *Heloniopsis tubiflora* (Korea) and *Xerophyllum tenax* (USA) to cover the gap between molecular data and explore “hot spots” for genomic events in Melanthiaceae. Comparative analysis of 23 complete cpDNA sequences revealed that there were different stages of deletion in the *rps16* region across the Melanthiaceae. Based on the partial or complete loss of *rps16* gene in cpDNA, we have firstly reported potential molecular markers for recognizing two sections (*Veratrum* and *Fuscoveratrum*) of *Veratrum*. Melathiaceae exhibits a significant change in the junction between large single copy and inverted repeat regions, ranging from *trnH_GUG* to a part of *rps3*. Our results show an accumulation of tandem repeats in the *rpl23-ycf2* regions of cpDNAs. Small conserved sequences exist and flank tandem repeats in further observation of this region across most of the examined taxa of Liliales. Therefore, we propose three scenarios in which different small repeated sequences were used during SSM events to generate newly distinct types of repeats. Occasionally, prior to the SSM process, point mutation event and double strand break repair occurred and induced the formation of initial repeat units which are indispensable in the SSM process. SSM may have likely occurred more frequently for short repeats than for long repeat sequences in tribe Parideae (Melanthiaceae, Liliales). Collectively, these findings add new evidence of dynamic results from SSM in chloroplast genomes which can be useful for further evolutionary studies in angiosperms. Additionally, genomics events in cpDNA are potential resources for mining molecular markers in Liliales.

## Introduction

Chloroplast genome sequences provide useful information for phylogenetic studies of higher level taxa, including families and orders (Zomlefer et al., 2001; Ji et al., 2006; Barrett et al., 2013; Kim and Kim, 2013; Kim et al., 2013, 2016a; Nguyen et al., 2013; Ruhfel et al., 2014). Structural changes such as small and large inversions, gene contents (duplication, triplication, and deletion), and pseudogenization have provided valuable resources for examining genome evolution among plants. Gene duplications have been reported in previous studies (Lee et al., 2007; Cai et al., 2008; Schmickl et al., 2009). Specifically, different copies of *trnF_GAA* were found in several genera of Brassicaceae (Schmickl et al., 2009). Additionally, repeated DNA sequences, which were assumed to have originated from different mechanisms such as gene conversion, unequal recombination, and slipped-strand mispairing (SSM), are main resources for genomic events of duplication, deletion, and rearrangement in chloroplast genomes (Levinson and Gutman, 1987; Cai et al., 2008; Huang et al., 2014; Sveinsson and Cronk, 2014).

Melanthiaceae is a family within the order Liliales that includes 16 genera divided into five tribes: Melanthieae (7 genera), Heloniadeae (3 genera), Parideae (3 genera), Chionographideae (2 genera), and Xerophylleae (1 genus) (Angiosperm Phylogeny Group, 2009, 2016; Govaerts, 2016; WCSP, 2016). Prior to its grouping within Liliales, these genera were classified into different orders of Dioscoreales and Melanthiales based on the morphological characteristics of their extrorse anthers and ovaries, and often with the presence of three styles (Rudall et al., 2000). The tribe Parideae, comprising *Paris, Psedotrillium*, and *Trillium*, was formerly treated as an independent family, Trilliaceae (Thorne, 1992; Takhtajan, 1997). However, based on molecular and morphological data, this tribe was later reclassified as monophyletic within Liliales (Chase et al., 2000; Angiosperm Phylogeny Group, 2009; Kim and Kim, 2013). Recently, Pellicer et al. (2014) identified the extreme variations in genome size and a significant reduction in the number of chromosomes in Parideae. The evolution of the chloroplast genome (cpDNA) has been investigated in *Veratrum patulum* (Do et al., 2013), *Chionographis japonica* (Bodin et al., 2013), *Paris verticillata* (Do et al., 2014), *Trillium* species (Kim et al., 2016b), and *Paris* sp. (Huang et al., 2016), which represent three tribes, Melanthieae, Chionographideae, and Parideae, respectively. Specifically, different numbers of *trnI_CAU* and repeat sequences in *rpl23-ycf2* regions and inversion was detected in tribe Parideae (Do et al., 2014; Huang et al., 2016; Kim et al., 2016b). The *rps16* gene was completely lost in *C. japoncica* and partially deleted in *V. patulum* (Bodin et al., 2013; Do et al., 2014). Collectively, these findings suggest that Melanthiaceae possess evidence of different genomic events in cpDNA. Nonetheless, these genomic events in Melanthiaceae have not been fully characterized because of the lack of cpDNA data.

In this study, we sequenced the complete chloroplast genomes of *Heloniopsis tubiflora* (GenBank Accession number KM078036) and *Xerophyllum tenax* (GenBank Accession number KM078035), representing the two unreported tribes of Heloniadeae and Xerophylleae, to cover the gap of cpDNA data within Melanthiaceae. Based on the complete cpDNA sequences, we characterized the differentiation, including gene loss, duplication, and fluctuation of IR-LSC boundary, among five tribes of Melanthiaceae. Then, we applied these features to create the first potential molecular marker for recognizing two sections of *Veratrum*. Additionally, we questioned the pattern and the mechanism of repeat's accumulation in *rpl23-ycf2* regions. Therefore, we sequenced this region among representatives from other families in Liliales and conducted comparative analyses of sequence data to (1) investigate the pattern of repeat's accumulation within *rpl23-ycf2* regions of examined species, and (2) propose hypothetical scenarios for the duplication process.

## Materials and methods

### Sample collection, DNA extraction, whole-genome sequencing, and assembly

Fresh leaves of *H. tubiflora* were collected in Deogyusan National Park, South Korea. Voucher specimens were deposited in the Herbarium of Gachon University (GCU). Dried leaves of *X. tenax* were obtained from the Forestfarm Plant Nursery (Williams, Oregon, USA). The plant materials used in this study were collected through the KNRRC (Medicinal Plants Resources Bank NRF-2010-0005790), supported by the Korea Research Foundation (resources provided by the Ministry of Education, Science and Technology in 2014). Total DNA was extracted using a DNAEasy Plant Mini Kit (Qiagen, Seoul, South Korea). These DNA samples were sequenced using the 454 system for *H. tubiflora* and the Hiseq2000 system for *X. tenax*. After removal of reads with ambiguous “N” bases, the remaining reads were trimmed with no more than a 5% chance of error per base before being mapped to the reference chloroplast genome sequences of *C. japonica* (Bodin et al., 2013) and *P. verticillata* (Do et al., 2014), to isolate chloroplast genome sequences using Geneious (Biomatters Ltd., Auckland, New Zealand). Based on the tribal relationship in Melanthiaceae (Kim et al., 2016a), we mapped the reads of *H. tubiflora* and *X. tenax* to cpDNA sequences of *C. japonica* and *P. verticillata*, respectively. The assembled reads were then extracted and reanalyzed in Geneious using the *De Novo* Assembly tool with the option of “no gaps or mismatches per read.” The consensus sequences generated from *De Novo* Assembly were used as references to reassemble raw reads. These steps were repeated until the complete cpDNA sequences were identified. Occasionally, gaps were present among chloroplast contigs. These remaining gaps were closed using the Sanger method with newly designed primers based on homologous sequences between the reads and reference sequences. Additionally, borders between the LSC, small single copy (SSC), and IR regions, as well as ambiguous regions (i.e., insertion and deletion events in coding regions and low coverage regions) were confirmed by Sanger sequencing methods. A total of 1,093,684 and 8,719,277 reads of *H. tubiflora* and *X. tenax* were generated, respectively. The results showed that cpDNA of *H. tubiflora* consisted of 37,973 (3.47%) out of 1,093,684 reads with a coverage rate of 19.2 x. For *X. tenax*, 196,299 reads (2.25%) belonged to chloroplast genome sequences with a coverage rate of 112.6 × over the cpDNA. The complete cpDNA sequences of *X. tenax* and *H. tubiflora* were deposited into GenBank under accession numbers KM078035 and KM078036, respectively (Table 1).

**Table 1:** **Comparison of the features of cpDNAs from five families of Liliales**.

**Family**	**Species**	**Accession number**	**Length (bp)**				**AT content (%)**	**GC content (%)**	**Protein coding genes**	**tRNAs**	**rRNAs**
			**Total**	**LSC region**	**SSC region**	**IR region**					
Melanthiaceae	*Paris verticillata*	KJ433485	157,379	82,726	17,097	28,373	62.4	37.6	81	30	4
	*Paris quadrifolia*	KX784051	157,097	83,772	18,287	27,924	62.3	37.7	81	30	4
	*Paris cronquisitii*	KX784041	157,710	84,502	18,316	27,446	62.7	37.3	81	30	4
	*Paris dunniana*	KX784042	157,984	84,482	18,364	27,569	62.8	37.2	81	30	4
	*Paris fargesii*	KX784043	157,518	84,549	18,311	27,329	62.7	37.3	81	30	4
	*Paris forrestii*	KX784044	158,345	84,396	18,671	27,639	62.7	37.3	81	30	4
	*Paris luquanensis*	KX784045	158,451	84,408	18,403	27,820	62.7	37.3	81	30	4
	*Paris mairei*	KX784046	157,891	84,420	18,361	27,555	62.7	37.3	81	30	4
	*Paris marmorata*	KX784047	157,566	84,221	18,301	27,522	62.7	37.3	81	30	4
	*Paris polyphylla var. chinensis*	KX784048	158,307	85,187	18,175	27,473	62.8	37.2	81	30	4
	*Paris polyphylla var. yunnanensis*	KX784049	157,547	84,224	18,319	27,502	62.7	37.3	81	30	4
	*Paris vietnamensis*	KX784050	158,224	84,794	18,360	27,535	62.8	37.2	81	30	4
	*Trillium maculatum*	KR780075	157,359	86,340	19,949	25,535	62.5	37.5	81	30	4
	*Trillium tschonoskii*	KR780076	156,852	83,981	19,869	26,501	62.5	37.5	81	30	4
	*Xerophyllum tenax*	KM078035	156,746	83,910	18,096	27,370	62.2	37.8	81	30	4
	*Heloniopsis tubiflora*	KM078036	157,940	84,840	18,018	27,541	62.5	37.5	81	30	4
	*Chionographis japonica*	KF951065	154,646	81,653	18,195	27,399	62.3	37.7	80	30	4
	*Veratrum patulum*	KF437397	153,699	83,372	17,607	26,360	62.3	37.7	81	30	4
Colchicaceae	*Colchicum autumnale*	KP125337	156,462	84,246	16,734	27,741	62.4	37.6	80	30	4
	*Gloriosa superba*	KP125338	157,924	85,012	16,786	28,063	62.4	37.6	81	30	4
Smilacaceae	*Smilax china*	HM536959	157,878	84,608	18,536	27,376	62.75	37.25	80	30	4
Liliaceae	*Lilium longiflorum*	KC968977	152,793	82,230	17,523	26,520	62.98	37.02	81	30	4
Alstroemeriaceae	*Alstroemeria aurea*	KC968976	155,510	84,241	17,867	26,701	62.74	37.26	80	30	4

*LSC, Large single copy; SSC, small single copy; IR, inverted repeat; bp, base pairs*.

### Genome annotation, comparison, visualization, and characterization of repeat sequences

**Figure 1:**
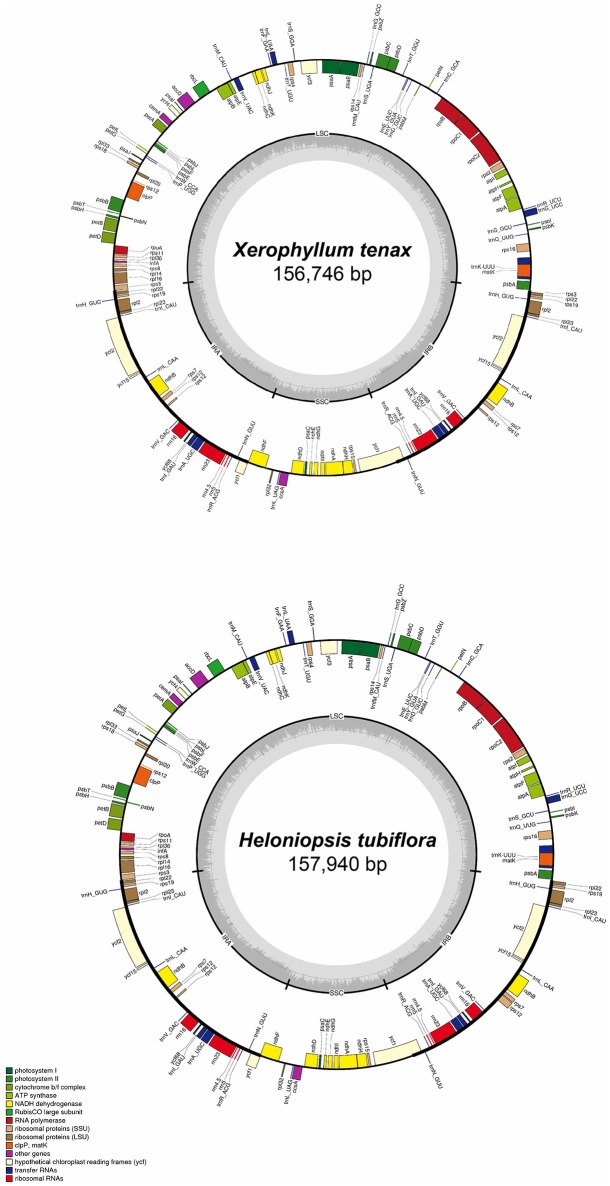
**Map of *Heloniopsis tubiflora* and *Xerophyllum tenax* chloroplast genomes**. Genes shown outside of the outer circle are transcribed counterclockwise, whereas those shown inside are transcribed clockwise. The thick lines in the small circles indicate the inverted repeat regions. The dark gray area in the inner circle indicates the CG content of the chloroplast genome. The colors represent different groups of genes in cpDNA. LSC, Large single copy; SSC, small single copy; IRA, inverted repeat region A; IRB, inverted repeat region B.

The complete cpDNA sequences of *H. tubiflora* and *X. tenax* were annotated using Geneious. All tRNA sequences were confirmed using the online web-based tool tRNAScan-SE (Schattner et al., 2005). The Mauve alignment, embedded in Geneious, was used to compare the 23 complete cpDNA sequences and to identify significant differentiation, including gene loss, duplication, and fluctuation of IR-LSC boundary with default settings (Darling et al., 2004). Genome maps were generated using OGDraw v1.2 (Lohse et al., 2013), followed by manual modifications. The maps of cpDNA sequences of *X. tenax* and *H. tubiflora*, which illustrate the genome structure and gene composition and order, were shown in Figure 1. The locations of repeat sequences were identified using Phobos (Mayer, 2006) with default settings.

### Characterization of *rps16* loss and junction of IR-LSC regions

After conducting alignment of complete cpDNA sequences using Geneious, we designed primer pairs for amplifying *trnK_UUU-trnQ_UUG* region, containing *rps16* (data not shown). Because of different PCR results of verifying *rps16* loss in tribe Melanthieae, primer pairs (rps16-F: 5′-GTCAATATGAATGTTGATAA-3′ and rps16-R: 5′-TTTTCTATTCCATACACATG-3′) were designed using Primer3 (Untergasser et al., 2012). The PCR profile consisted of denaturation at 94°C for 3 min followed by 35 cycles at 94°C for 1 min, 54°C for 1 min, and 72°C for 1 min, with a final extension at 72°C for 7 min. These newly designed primer pairs were applied for *Veratrum* species of two sections including *Veratrum* (*V. oxysepalum*—JK Hong 015; *V. lobelianum*—Chase 19618; *V. grandiflorum*—KUN0303565) and *Fuscoveratrum* (*V. versicolor*—GCU1411788; *V. maackii*—KWU03358; *V. nigrum*—GCU121205181). All PCR products were purified using the MEGAquick-spin Total Fragment DNA Purification Kit (iNtRON Biotechnology, Seoul, Korea) and sequenced using the BigDye Terminator Cycle Sequencing Kit (Applied Biosystems, Foster City, CA, USA) according to the manufacturer's instructions. These sequences were assembled and annotated in Geneious.

For identifying junction of IR/LSC regions, we designed primer pairs for *rpl2-psbA* and *rpl2-rps3* regions (data not shown). The PCR products were sequenced and then annotated in Geneious. After getting annotation results, these sequences were aligned to identify the border among examined species.

### Confirmation of duplication events in liliales

To confirm the duplication patterns in Liliales, we sampled 68 taxa of 9 families within Liliales, except Corsiaceae which contains mycoheterotrophic species (Table 2). Total genomic DNA was extracted from dried leaves using a modified CTAB method based on Doyle and Doyle (1987). Primer pairs that amplify the entire *rpl23-ycf2* IGS were applied with the same PCR profile from the description of Kim et al. (2016b). The further steps for PCR product's treatment were conducted identically as the description above.

**Table 2:** **List of the surveyed taxa with the length of *rpl23-ycf2* intergenic space sequences, number of *trnI_CAU* copies, number of repeats and the hypothetical scenarios of repeat's accumulation**.

**Species**	**Voucher (Herbarium) or accession number or references**	**Length of *rpl23-ycf2* IGS (bp)**	**Number of *trnI_CAU* copy**	**Length of IGS of *rpl23-trnI* and *trnI-ycf2***	**Total number of repeat units (>=18 bp)**	**Hypothetical scenarios**
***Melanthiaceae***						
*Paris verticillata*	S.C Kim 009 (GCU)	591	3	172/67	3	III-B
*Paris quadrifolia*	Ian Christie—SRGC	591	3	172/67	3	III-B
*Paris incompleta*	BONN 22706	640	3	164/92	3	≠
*Paris japonica*	Chase 29052 (KEW)	818	2	159/146	2#	≠
*Paris dulongensis*	KUN 0301542	724	1	159/491	16	III-A
*Paris fargesii*	Kim et al., 2016b	708	1	159/475	16	III-A
*Paris mairei*	Kim et al., 2016b	612	1	159/379	12	III-A
*Paris luquanensis*	Kim et al., 2016b	636	1	159/403	13	III-A
*Paris rugosa*	Kim et al., 2016b	612	1	159/379	12	III-A
*Paris dunniana*	Kim et al., 2016b	420	1	159/187	4	III-A
*Paris thibetica*	Kim et al., 2016b	485	1	159/252	7	III-A
*Parisaxialis*	Kim et al., 2016b	501	1	159/268	6	III-A
*Paris vietnamensis*	Kim et al., 2016b	500	1	159/267	6	III-A
*Paris polyphylla* var. *chinensis*	Kim et al., 2016b	523	1	159/290	12	III-A
*Paris polyphylla* var. *polyphylla*	Kim et al., 2016b	612	1	159/290	8	III-A
*Paris polyphylla* var. *stenophylla*	Kim et al., 2016b	636	1	159/403	13	III-A
*Pseudotrillium rivale*	Kim et al., 2016b	299	1	164/61	0	–
*Trillium undulatum*	Kim et al., 2016b	307	1	165/68	0	–
*Trillium decumbens*	NC_027282	402	1	205/123	0	–
*Trillium cuneatum*	NC_027185	378	1	210/94	0	–
*Trillium smalii*	Kim et al., 2016b	447	1	173/200	7	III-A
*Trillium tschonoskii*	KR780076	483	1	240/169	7	III-A
*Trillium flexipes*	Kim et al., 2016b	637	1	173/390	18#	III-A^*^
*Trillium simile*	Kim et al., 2016b	651	1	173/404	19#	III-A^*^
*Trillium rugelii*	Kim et al., 2016b	665	1	169/422	20#	III-A^*^
*Trillium erectum*	Kim et al., 2016b	669	1	173/422	20#	III-A^*^
*Trillium underwoodii*	Kim et al., 2016b	602	2	205/113	2#	II^*^
*Trillium chloropetalum*	Kim et al., 2016b	581	2	205/102	2	II
*Trillium luteum*	Kim et al., 2016b	565	2	210/94	2	II^*^
*Trillium sessile*	Kim et al., 2016b	565	2	210/94	2	II^*^
*Trillium maculatum*	KR780075	569	2	214/94	2	II^*^
*Trillium govanianum*	Kim et al., 2016b	612	3	164/65	3	I
*Xerophyllum tenax*	KM078035	306	1	164/68	0	–
*Xerophyllum asphodeloides*	Kim et al., 2016b	306	1	164/68	0	–
*Chionographis japonica*	KF951065	302	1	160/68	0	–
*Heloniopsis tubiflora*	KM078036	302	1	160/68	0	–
*Veratrum patulum*	KF437397	304	1	157/73	0	–
Anticlea elegans^*^	RBGE 19560436A	322	1	157/91	2	–
Stenanthium densum^*^	RBGE 19661391A	322	1	157/91	2	–
*Toxicoscordion micranthus*	RBGE 19951902A	304	1	157/73	0	–
*Schoenocaulon coricifolium*	Chase 1852	304	1	157/73	0	–
*Zigadenus glaberrimus*	Chase 153	299	1	157/68	0	–
***Liliaceae***						
*Lilium longiflorum*	KC968977	307	1	165/68	0	–
*Fritillaria cirrosa*	NC_024728	307	1	165/68	0	–
*Calochortus venustus*	GCU06200	307	1	165/68	0	–
*Tricyrtis macropoda*	GCU06199	307	1	165/68	0	–
*Gagea triflora*	TUT33042	307	1	165/68	0	–
*Erythronium japonicum*	GCU05168	307	1	165/68	0	–
*Tulipa sylvestris*	Avon Bulbs 131-37B5	307	1	165/68	0	–
*Clintonia udensis*	Hong 053	307	1	165/68	0	–
*Streptopus ovalis*	KWU01411	307	1	165/68	0	–
***Smilacaceae***						
*Smilax china*	HM536959	307	1	165/68	0	–
*Smilax nipponica*	Hong 008	307	1	165/68	0	–
*Smilax glyciphylla*	Kim and Bodin 2013-2 (GCU)	307	1	165/68	0	–
*Heterosmilax china*	C.X Fu (KUN)	307	1	165/68	0	–
***Philesiaceae***						
*Philesia magellanica*	CL0BR19850151 Botanical Garden Maise, Belgium	307	1	165/68	0	–
***Rhipogonaceae***						
*Rhipogonum scandens*	F2010036. Garden of Auckland, New Zealand	307	1	165/68	0	–
***Colchicaceae***						
*Colchicum autumnale*	KP126337	298	1	165/59	0	–
*Gloriosa superba*	KP125338	307	1	165/68	0	–
*Wurmbea burtii*	Peter Brownless, Royal Botanic Garden Edinburgh	302	1	160/68	0	–
*Tripladenia cunninghamii*	Kim and Bodin 2013-1 (GCU)	299	1	157/68	0	–
*Uvularia grandiflora*	Floden et al., 1246	307	1	165/68	0	–
*Disporum smilacinum*	S.C. Kim 05267 (GCU)	307	1	165/68	0	–
***Alstroemeriaceae***						
*Alstroemeria aurea*	KC968976	307	1	165/68	0	–
*Bomarea edulis*	KM233641	307	1	165/68	0	–
*Luzuriaga radicans*	KM233640	307	1	165/68	0	–
***Petermanniaceae***						
*Petermannia cirrosa*	Kim and Bodin 2013-3(GCU)	307	1	165/68	0	–
***Campynemataceae***						
*Campynema lineare*	NC_026785	312	1	170/68	0	–

KUN, Herbarium of Kunming Institute of Botany; TUT, Daejeon University Herbarium; GCU, Gachon University Herbarium; KEW, the Royal Botanic Gardens Kew; SRGC, Scottish Rock Garden Club. The dagger (≠) indicates complex duplication events which are not able to be divided into three scenarios. The number sign (#) represent the species which possess two type of repeat sequences. The asterisk (^*^) shows the species which have direct repeats. The dashes (–) mean the species without hypothetical scenarios of repeat's accumulation in comparison with other species.

## Results

### Features of cpDNA among five families of liliales and five tribes of melanthiaceae

**Figure 2:**
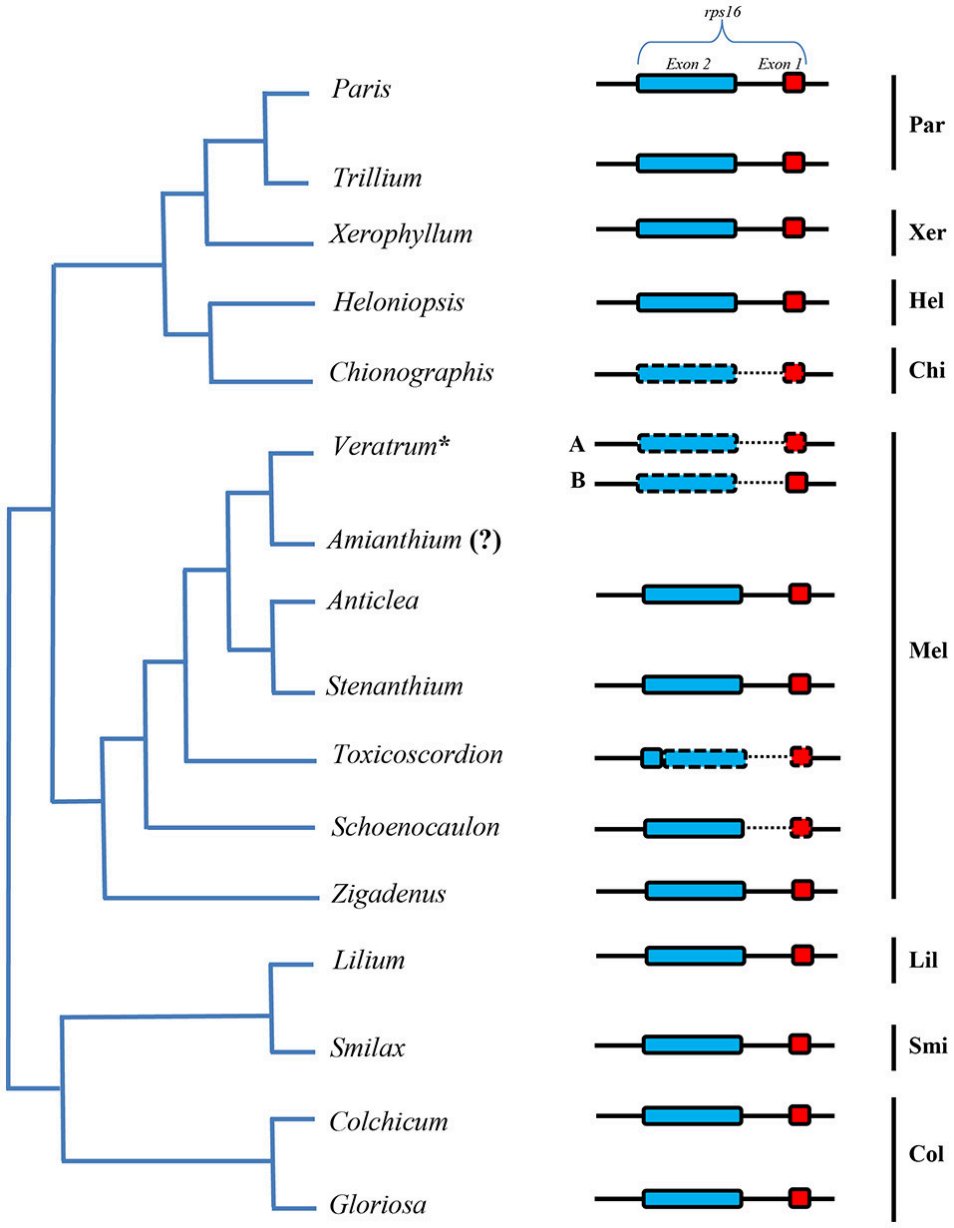
**Comparison of *rps16* among species of Melanthiaceae and other families**. The simplified phylogenetic tree was based on Kim et al. (2016a). The dashed line box indicates the lost sequences and the dotted line showed the significantly changed sequences in comparison with others. Par, Parideae; Xer, Xerophylleae; Hel, Heloniadeae; Chi, Chionographideae; Mel, Melanthieae; Lil, Liliaceae; Smi, Smilacaceae; Col, Colchicaceae. The question mark (?) means missing data. The asterisk shows the genus which has two types of *rps16* loss (A) completely lost and (B) partially lost.

**Figure 3:**
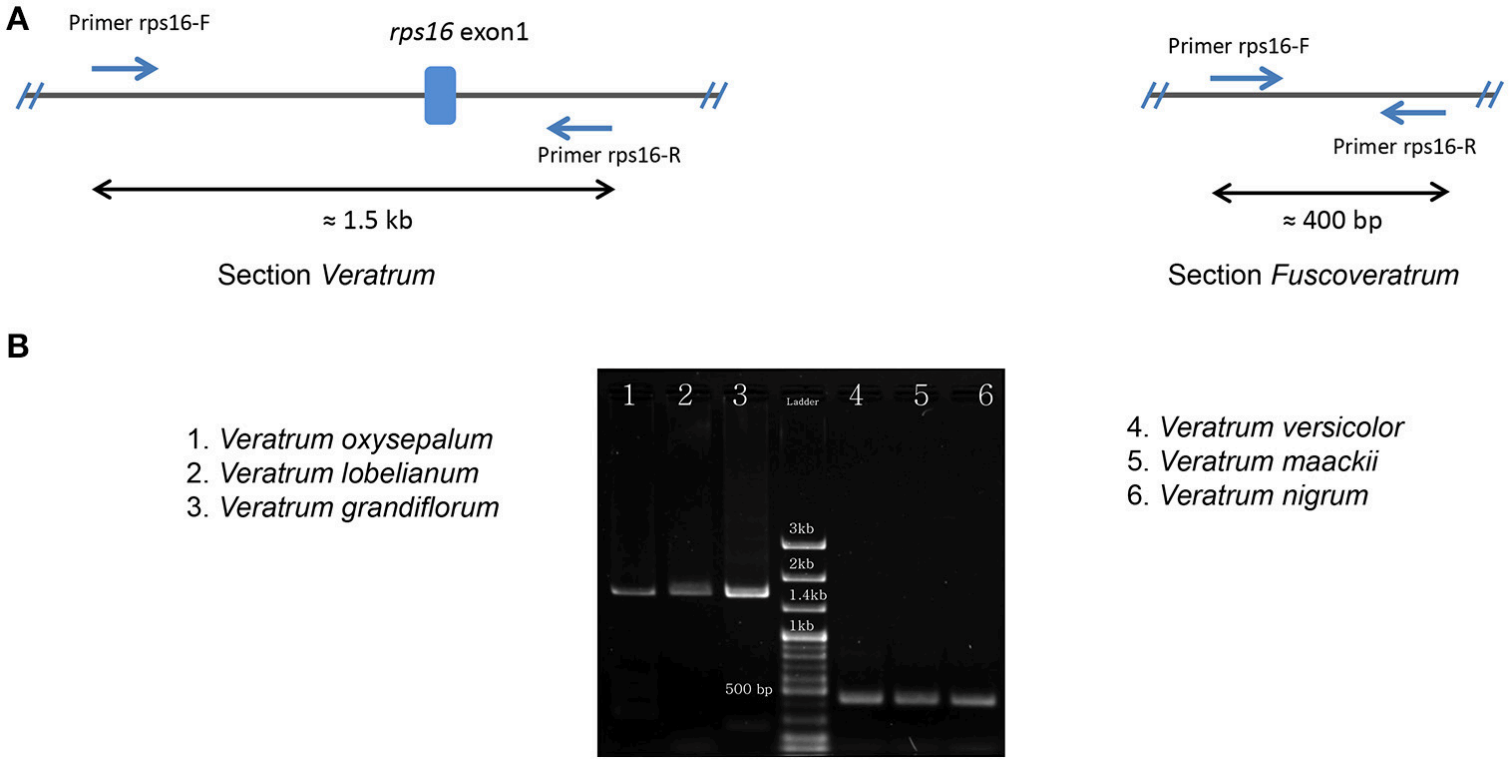
**Confirmation of *rps16* gene loss in *Veratrum* species. (A)** The design of primers (Forward primer: rps16-F; Reverse primer: rps16-R) which cover the whole coding sequences of *rps16* based on the cpDNA sequence of *V. patulum* (Accession number KF437397) and their positions in two types of *rps16* gene loss. Expected lengths are ~1.5 kb in section *Veratrum* and 400 bp in section *Fuscoveratrum*. **(B)** Results of PCR among species of *Veratrum*. The section *Veratrum* includes *V. oxysepalum, V. Lobelianum*, and *V. grandiflorum*. The section *Fuscoveratrum* includes *V. versicolor, V. maackii*, and *V. nigrum*.

The lengths of circular double-stranded DNA molecules differed among the families, ranging from 152,793 bp (*Lilium longiflorum*, Liliaceae) to 158,451 bp (*Paris luquanensis*, Melanthiaceae, Table 1). In Melanthiaceae, *V. patulum* (tribe Melanthieae) possesses the smallest cpDNA whereas the biggest cpDNA belongs to *Paris* species (tribe Parideae, Table 1). Although, the lengths varied, the AT and GC contents were relatively stable among the observed taxa (Table 1). Most of the examined species have 81 protein-coding genes, 30 tRNAs and 4 rRNAs in cpDNA sequences. However, there were 80 protein-coding genes in *C. japonica, Colchicum autumnale*, and *Alstroemeria aurea* because of the complete loss of *rps16* in *C. japonica* and the deletion of whole region of *ycf15* in *C. autumnale* and *A. aurea*. Comparative genomic analysis among five representative taxa of Melanthiaceae revealed that the deletion of *rps16* was only found in tribes Chionographideae and Melanthieae. Further investigation on *rps16* among genera of tribe Melanthieae revealed that the loss of *rps16* was not common and only found in *Veratrum, Toxicoscordion*, and *Schoenocaulon* (Figure 2). Specifically, exon 1 of *rps16* was deleted in *Schoenocaulon* whereas a part of exon 2 of this gene (47 bp) remained in *Toxicoscordion*. In contrast to complete loss of *rps16* in *C. japonica*, only exon 2 of this gene was deleted in *V. patulum*, suggesting that there were different stages of this event in *Veratrum* genus which was divided into two sections: *Veratrum* and *Fuscoveratrum*. To track the loss of *rps16*, one primer pairs which covered the whole coding regions of *rps16* was designed and applied for *Veratrum* species (Figure 3A). As expected, the PCR results revealed that there were two types of deletion of *rps16* in *Veratrum* (Figure 3B). The first type was found in section *Veratrum* of which exon 2 of *rps16* was lost. Remaining of exon 1 of *rps16* resulted in a PCR product of ~1.5 kb in three examined taxa of section *Veratrum* (*V. oxysepalum, V. lobelianum*, and *V. grandiflorum*; Figures 3A,B). In contrast, the deletion of whole coding region of *rps16* in section *Fuscoveratrum* caused a 400 bp-PCR product in *V. versicolor, V. maackii*, and *V. nigrum* (Figures 3A,B). In Liliaceae, Smilacaceae, Altroemeriaceae, and Colchicaceae, the intact coding sequence of *rps16* was found (Table 1, Figure 2).

**Figure 4:**
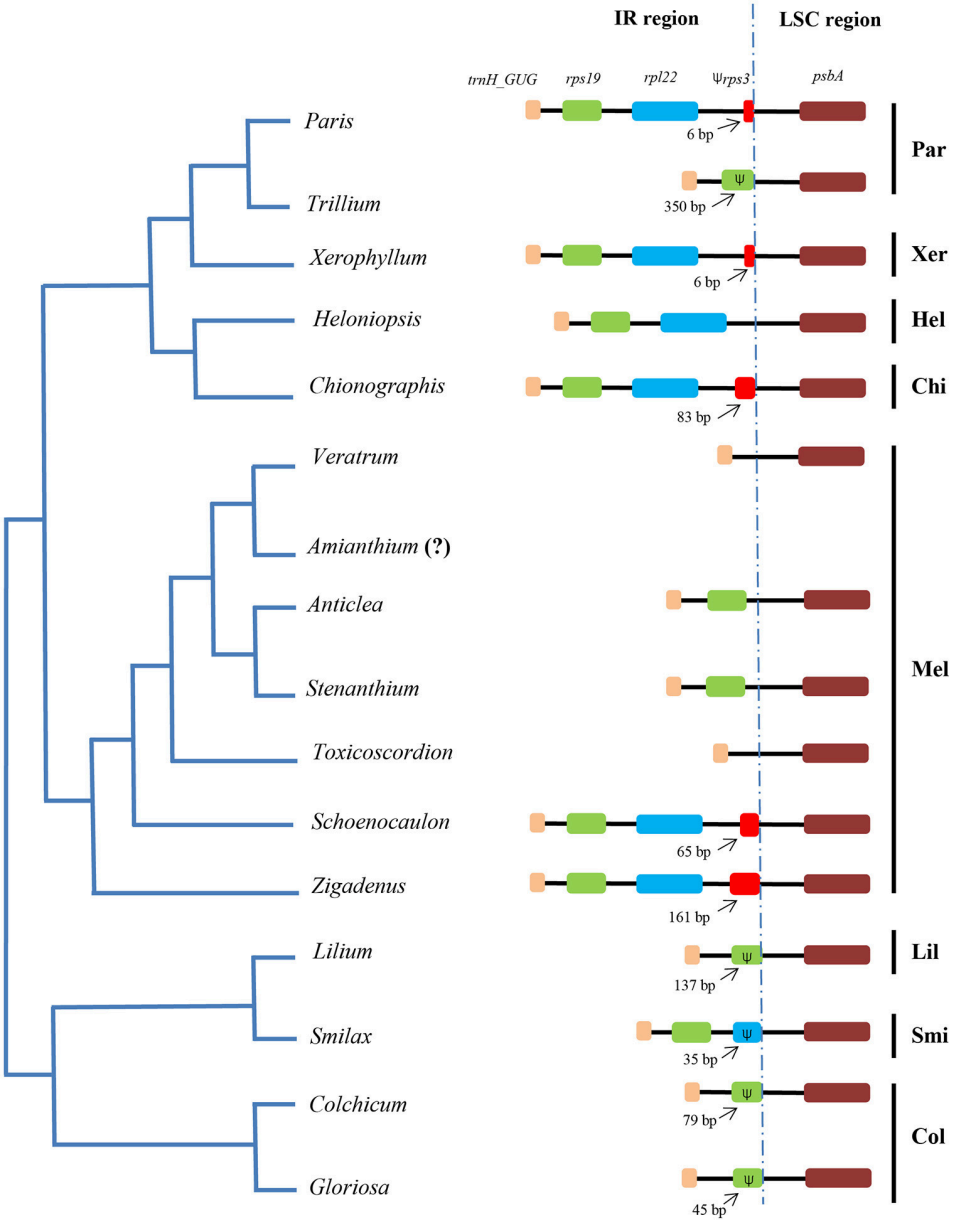
**Comparison of borders between IR and LSC regions among species of Melanthiaceae and other families**. The simplified phylogenetic tree was based on Kim et al. (2016a). The dotted line indicates the junction of IR-LSC regions. The (Ψ) represents pseudogenes. Par, Parideae; Xer, Xerophylleae; Hel, Heloniadeae; Chi, Chionographideae; Mel, Melanthieae; Lil, Liliaceae; Smi, Smilacaceae; Col, Colchicaceae. The question mark (?) means missing data.

Sequences flanking the LSC/IR junction were compared between other taxa of the Liliales (Figure 4). The IR/LSC borders varied among the taxa. Specifically, the IR/LSC borders located in coding region of *rps19* in Liliaceae and Colchicaceae. Meanwhile, it expanded to a part of *rpl22* in Smilacaceae (Figure 4). In Melanthiaceae, it occurred in the *trnH_GUG*/*rps19* intergenic spacer in *Veratrum* and *Toxicoscordion*. However, in other taxa, it expanded into a part of *rps19* (350 bp in *Trillium*), into the *rps19/rpl22* intergenic spacer (IGS; *Anticlea* and *Stenanthium*), into the *rpl22*/*rps3* IGS (*Heloniopsis*), and into a section of *rps3* (6 bp in *Xerophyllum* and *Paris*; 83 bp in *Chionographis;* 65 bp in *Schoenocaulon;* 161 bp in *Zigadenus*). Compared to other tribes, Melanthieae possessed a wide range of IR/LSC junctions (Figure 4).

### Accumulation of repeat sequences in tribe parideae

**Figure 5:**
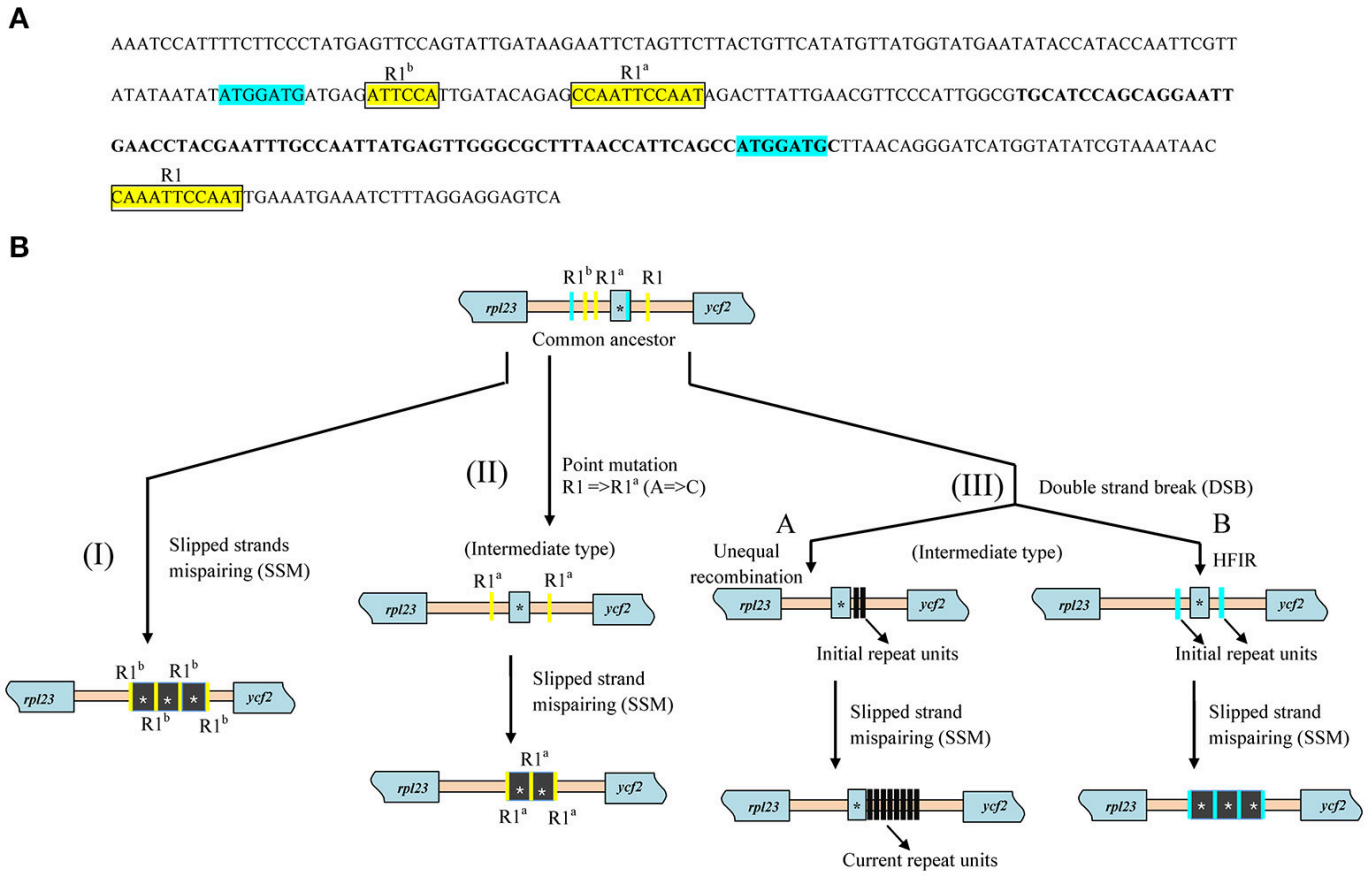
**Conserved repeated sequences in *rpl23-ycf2* IGS among Liliales species and hypothetical pathways of repeat's accumulation. (A)** The representative sequence of *rpl23-ycf2* IGS extracted from *Campynema lineare* (Accession number NC026785) and positions and two groups of conserved repeats in Liliales. Blue shaded and squared yellow shaded sequences represent two groups of repeats. The letters of R1, R1a, and R1b above sequence indicate variety of repeats. The bold letters indicate the coding region of *trnI_CAU* in *rpl23-ycf2* IGS sequence. **(B)** Hypothetical scenarios for formation of repeats. The blue and yellow bars represent repeat units. Black bars indicate new generated repeats. HIFR stands for homology facilitated illegitimate recombination. Asterisks showed the *trnI_CAU* sequence which can be included in repeat units.

Further investigation of repeat sequences showed that the IGS between *rpl23* and *ycf2* containing *trnI_CAU* was extremely variable in length, ranging from 299 to 818 bp among *Paris* and *Trillium*, while a more stable length was detected in other species (Table 2). In *Paris*, the *trnI-ycf2* IGS was ranged from 67 to 491 bp. As is the case in *Paris, Trillium* has varying lengths of the *trnI*–*ycf2* IGS (from 68 to 422 bp). Notably, nearly all species in other Liliales families have equal lengths of the *trnI-ycf2* IGS (68 bp). The length variation in the *rpl23-ycf2* IGS of tribe Parideae can be attributed to the presence of tandem repeat sequences (Table 2, Supplementary Data S1). The results of repeat analysis in *rpl23-ycf2* IGS regions among Liliales species showed that accumulation of repeat occurred only in tribes Parideae and Melanthieae of Melanthiaceae. Additionally, the number of repeat units was different among examined species (Table 2). In *Paris* species, this region contained a ranging copy number from 2 to 16 whereas the number of copy varied from 2 to 20 in *Trillium* taxa (Table 2, Supplementary Data S1). Also, the length of these repeats was different in both genera, ranging from 24 to 155 bp in *Paris* and from 18 to 209 bp in *Trillium* (Supplementary Data S1). Additionally, we observed upstream and downstream of repeats because of the important role of initial repeats in SSM mechanism. As a result, we found two groups of small conserved repeated sequences in most of the surveyed taxa (Figure 5A, Supplementary Data S1). In the first group, there were two 7 bp—direct repeats which were located upstream and within the coding sequence of *trnI_CAU*. In the rest group, there was a cluster of direct repeats including R1 (5′-CAAATTCCAAT-3′), R1^a^ (5′-CCAATTCCAAT-3′), and R1^b^ (5′-ATTCCA-3′).

## Discussion

### Comparative characteristics of cpDNA among melanthiaceae species and its implication

The cpDNA structures of representative species of Melanthiaceae consist of typical double-stranded DNA molecules and are highly conserved, as reported in previous angiosperm cpDNA studies (Palmer, 1991; Yang et al., 2010; Liu et al., 2012; Huang et al., 2013, 2014, 2016; Kim and Kim, 2013; Luo et al., 2014; Nguyen et al., 2015). In this study, length variations were identified among the Melanthiaceae taxa (Table 1). The longer sequences of cpDNA were found in *Paris, Trillium, Xerophyllum*, and *Heloniopsis* species which possessed either repeat units in *rpl23-ycf2* regions or expansion of IR/LSC border. Although, *C. japonica* has the expansion of IR/LSC junction to *rps3* (83 bp), the loss *of rps16* caused a shorten length of its cpDNA. Therefore, it is suggested that the length variations within Melanthiaceae could have been led by the deletion and duplication of genes, as well as the expansion of IR regions. The comparative analysis among five families of Liliales revealed a notable variety of length and different losses of genes in cpDNA (Table 1). However, further studies, which cover all 10 families, should be conducted to investigate the overall trends of genomic events in Liliales.

The *rps16* gene, encoding ribosomal protein S16, is commonly detected in the plant chloroplast genomes. However, the loss of this gene was also recorded in different taxa including *Connarus, Epifagus, Pinus, Viola, Fagus*, legume species, and etc. (Downie and Palmer, 1992; Doyle et al., 1995). For understanding this loss, it was proposed that the *rps16* gene was transferred to the nucleus and its protein product was able to target both chloroplast and mitochondria in the case of *Medicago truncatula* and *Populus alba* (Ueda et al., 2008). Additionally, deletion of *rps16* was found in a moss species of *Physcomitrella patens* subsp. *patens* (Sugiura et al., 2003). These results suggest that the transfer event of *rps16* occurred independently at the early divergence of plants. It was lost in *C. japonica* and partially or completely deleted in *V. patulum, S. densum, and T. micranthus* among the Melanthiaceae; therefore, ribosomal protein S16 was predicted to be untranscribed and untranslated from cpDNA. However, this deficiency could be compensated from nuclear *rps16* products as described in a previous study (Ueda et al., 2008). In contrast to the deletion of exon 2 and complete loss of *rps16* in *Chionographis* and *Veratrum*, the deletion of exon 1 and remains of a piece of exon 2 were recorded in *Schoenocaulon* and *Toxicoscordion*, respectively. Additionally, 22 out of 26 species of *Schoenocaulon* are endemic from the Southern of United States of America to Peru (Zomlefer and Judd, 2008). Therefore, this genomic feature might contribute to investigating the evolution of cpDNA in this genus. Further studies which cover all species of *Schoenocaulon* and *Toxicoscordion* should be conducted to clarify the overview of this feature in the tribe Melanthieae. Furthermore, two types of *rps16* deletions were found in two sections of *Veratrum* which were distinguished by characteristics of leaf, style, and sheath of stem base (Chen and Takahashi, 2000; Zomlefer et al., 2003; Figure 3). Previously, genomic events in chloroplast genome sequences were specifically detected in some species and could be molecular markers. For example, the inversion of the *trnV_UAC-atpB* region was only detected in species of *Trillium* subgenus *Phyllantherum* of Melanthiaceae and the loss of *ycf15* was observed in tribe Colchiceae of Colchicaceae (Nguyen et al., 2015; Kim et al., 2016b). In this study, based on the finding of partial or complete loss of *rps16*, we provide the first potential molecular maker for recognizing two sections among *Veratrum* (Figure 3). From these results, it is likely that genomic events in chloroplast genomes are effective for making molecular markers and reflect the phylogeny among Liliales taxa.

In general, IR expansion affects length variation in cpDNA. For example, the expansion of the IR region (36,501 bp) into *psbB* in *Mahonia bealei* cpDNA led to an increased total genome length (164,792 bp; Ma et al., 2013). In Melanthiaceae, the IR/LSC junctions were also variable (Figure 4). This variability affected the total length of the cpDNA region. For instance, the IR/LSC junction expansion from *trnH_GUG* into *rps3* resulted in an increased length of the IR region from 26,360 bp in *V. patulum* to 28,373 bp in *P. verticillata* (Table 1). Wang et al. (2008) suggested that the IR/LSC junctions in the Liliales taxa contained the *trnH_GUG*—*psbA* cluster, but variable patterns of junction existed in the order Liliales (Figure 4). Within the same family of monocots, IR/LSC junctions contained similarities; for example, the boundaries located in the *rps19* and *rpl22* genes of the Arecaceae and Orchidaceae, respectively (Huang et al., 2013; Luo et al., 2014). A similar trend was observed in dicots species of the Araliaceae, in which a common IR/LSC boundary was detected in the *rps19* gene (Li et al., 2013). In contrast, the border of IR/LSC varied among Melanthiaceae in which the IR region was expanded from *trnH_GUG* into a part of *rps3* (Figure 4). Significantly, there were three different borders in tribe Melanthieae (Figure 4). The unique expansion into 161 bp of *rps3* might be a potentially molecular marker for monotypic species—*Z. glaberrimus* (Figure 4).

### Hypothetical scenarios for dynamic SSM events in cpDNA

Previously, the DSB mechanism induced recombination in *Chlomydomonas reinhardtii* cpDNA (Dürrenberger et al., 1996). Kwon et al. (2010) reported the DSB repair pathways from both microhomology and no homology in *Arabidopsis*. Additionally, cpDNA sequences typically contain two inverted repeat regions which can be reversely used as a template for repairing the break of DNA through recombination. Tandem repeats ranging from 6 to 33 bp in IR region was discovered in *Oenothera* species (Onagraceae, Myrtales) (Blasko et al., 1988; Nimzyk et al., 1993; Sears et al., 1996), and tandem repeats comprising a 29-bp sequence have been found in the *rps8-rpl14* IGS of the LSC region of *Oenothera* (Wolfson et al., 1991). The copy correction of IR regions after imprecise alignment, replication slippage, and recombination have also been proposed as a mechanism for the accumulation of tandem repeats in *Oenothrea* cpDNA (Blasko et al., 1988; Wolfson et al., 1991; Sears et al., 1996). Recently, Massouh et al. (2016) surveyed and found spontaneous mutants in chloroplast genomes of *Oenothera* which were mostly caused by the replication slippage events. SSM was believed to be a major factor for DNA evolution (Levinson and Gutman, 1987). Although, results of SSM were previously reported in cpDNA of angiosperms, there have not been records of utilization of different small conserved repeats in the same region of cpDNA for generating newly repeated sequences. In this study, due to the presence of the conserved regions which flanked tandem repeats, we proposed three different patterns for generating the repeated sequences among Parideae taxa (Figure 5A). In the first scenario (I), the R1^b^ sequence was utilized through SSM mechanism to form three tandem repeats of 164 bp in *Trillium govanianum* which includes the whole *trnI_CAU* sequence (Supplementary Data S1). Within the second pathway (II), prior to the process of SSM, a point mutation which changed the adenine base to cytosine base to create a perfect direct repeat between R1 and R1^a^ occurred. The present of direct repeat (R1^a^) induced SSM process which resulted in formation of two repeats in *Trillium* taxa. Generally, in the SSM mechanism, initial repeats play an important role. Therefore, in the third case (III), we proposed the formation of initial repeats through the double-strand break (DSB) repair mechanism (Figure 5B). Specifically, two repair mechanisms may be involved in this case due to the difference in repeat contents among species. First, in the III-A subcase, unequal recombination occurred downstream of *trnI_CAU* and induced the formation of initial repeats which were employed in SSM process. Meanwhile, in the second subcase (III-B), repairing mechanism of DSB through homology facilitated illegitimate recombination (HFIR) occurred based on direct repeat sequences of 7 bp (5′-ATGGATG-3′) to create a longer 16 bp- repeat unit (5′-ATGGATGCTTAACAGG-3′) which was assumed to be an initial repeat unit for SSM event. Because of the different initial repeat units, SSM events occurred and resulted in newly distinct types of repeat sequences in both *Paris* and *Trillium* species (Figure 5B, Table 2, Supplementary Data S1). Albeit the sequence data supported our hypothetical scenarios, there was not essential evidence of *in vivo* experiment in this study. However, GuhaMajumdar et al. (2008) previously attempted to trace replication slippage *in vivo* and successfully confirmed this event from results of deletion and duplication in *C. reinhardtii* and *Escherichia coli*. Although, this study employed only one type of short tandem repeat, it fundamentally supported the reliability of three hypothetical scenarios in our study. Further studies, which use more types of small sequence repeats in the same region, should be conducted to provide substantial evidence for our hypothesis.

Recently, Kim et al. (2016b) used the number of *trnI_CAU* to classify the type of duplication events across Parideae. This classification was incongruent with infrageneric circumscription of *Paris* members, but not for *Trillium* species. In contrast, in terms of the origin of repeat sequences, there were no relationships between the classification within tribe Parideae and mechanisms of repeat's accumulation. For instance, in *Paris*, the formation of repeats could be explained by the (III) scenario, except *P. incompleta* whose repeats have likely arisen from the (I) pathway followed by point mutation events and *P. japonica* which reflected complex duplication processes (Table 2, Supplementary Data S1). In *Trillium*, all three pathways can be found. For example, the (I) scenario was recorded only in *T. govanianum*. Meanwhile, the (II) and (III-A) pathways could be found in subgenus *Phylantherum* and subgenus *Trillium*, respectively. Moreover, repeats were not found in *rpl23-ycf2* IGS of *T. undulatum* (Subgenus *Trillium*), or in *T. decumbens* and *T. cuneatum* (subgenus *Phylantherum*). These findings suggested that SSM events occurred independently across the tribe Parideae. Additionally, the number of <24 bp—repeat units was more abundant than those of over 24 bp in length, suggesting that that SSM occurred more frequently for short repeats than for long repeat sequences in the tribe Parideae (Melanthiaceae, Liliales). In *Anticlea elegans* and *S. densum*, two repeats (19 bp) were found (Table 2), suggesting that the accumulation of repeats may also occur in tribe Melanthieae, which is composed of 7 genera and 78 species of Melanthiceae.

Although, sequence data provided evidence for different scenarios of SSM and its independence, there was not enough evidence regarding the alternation of initial repeats during the SSM process in Parideae. Notably, small conserved units were found in most of the examined taxa; however, within Liliales, the repeats in the *rpl23-ycf2* IGS were mainly present in the tribe Parideae of Melanthiaceae. Therefore, variation within this region may be due to a unique genomic event in Parideae. Previous studies have found diverse genome sizes among Melanthiaceae (Pellicer et al., 2014). In contrast to the trend of reduced genome size in other tribes, Parideae exhibit significant increases in chromosome size and possess the largest nuclear genome in Melanthiaceae. This trend can also been seen in the patterns of repeats between Parideae and other tribes. It is likely that the causes of chromosome changes in Parideae might be related to the accumulation of repeats within this tribe. More studies should be conducted to shed light on the significance of these two unique features of the genomes of Parideae. Additionally, accumulation of repeat sequences was also found in *rpl23-ycf2* IGS of monocots such as *Acorus calamus* (Accession number NC_007407), *Sagittaria lichuanensis* (Accession number NC_029815), *Anomochloa marantoidea* (Accession number NC_014062), *Eustrephus latifolius* (Accession number KM_233639), *Curcuma roscoeana* (Accession number KF_601574), and *Musa acuminata* subp *malaccensis* (Accession number HF677508; Data not shown), suggesting that the *rpl23-ycf2* IGS may be one of the “hot spots” for genomic events in angiosperm species.

## Conclusions

In conclusion, comparative analysis of cpDNA in Melanthiaceae revealed that genomic events including pseudogenization, duplication, and deletion in the chloroplast genome are precise sources for mining molecular marker in plants. Specifically, gene loss events of *rps16* were potentially valuable molecular data for identifying two sections of the *Veratrum* species. Melanthiaceae also exhibits a significant change in junctions between LSC and IR regions. Additionally, we provided the first evidence of different employments of small repeat sequences for SSM in chloroplast genomes of monocots species. Though the origin of these differences remains unclear, these data highlight the dynamic molecular evolution in chloroplast genomes. With the increasing number of complete organelle genomes, these patterns could be detected in other species and be useful references for tracing genomic evolution among plants.

## Author contributions

HDKD carried out the genomic experiment and drafted the manuscript. HDKD and J-HK participated in the design of the study and revised the manuscript. All authors read and approved the final manuscript.

## Funding

This work was supported by the National Research Foundation of Korea (NRF) Grant Fund (MEST 2010-0029131) and Scientific Research of Korea National Arboretum (KNA) Grant Fund (KNA 1-2-13, 14-2).

### Conflict of interest statement

The authors declare that the research was conducted in the absence of any commercial or financial relationships that could be construed as a potential conflict of interest.
