# Supplementary material for: A Dynamic Tandem Repeat in Monocotyledons Inferred from a Comparative Analysis of Chloroplast Genomes in Melanthiaceae
Source: Front Plant Sci. 2017 May 22;8:693. doi: 10.3389/fpls.2017.00693 (PMC5438981; doi:10.3389/fpls.2017.00693)
Supplement: Supplementary file 1 [file DataSheet1.PDF]

Supplementary Data S1. Sequences of *rpl23-ycf2* IGS among *Paris*, *Trillium* and other species.

The grey-shaded sequences show the conserved sequences among species. The light grey-shaded sequences show the conserved sequences which have point mutations in comparison with grey-shaded sequences. The red and green braces indicate start and end positions of repeat units and show two different repeat units within a specie. The bold sequences represent the *trnI_CAU*. The light blue letters mean the different bases among repeats in the same taxon.

>*Paris verticillata* and *Paris quadrifolia* – The blue lettering indicates a base difference between two species; number of tandem repeats: 3 (139 bp).
AAATCCATTTTCTTCCCTATGAGTTCCAGTATCGATAAGAATTCTAGTTCTTACTGTTCATATGTTATGGTATGAATATA
CCATATCAATTCGTTATGT{ATGGATGCTTAACAGGAATCATCGTAAATAAAATAACCAAATTCCAATAGACTTATTGA
ACGTTCCATTGGCG**TGCATCCAGCAGGAATTGAACCTACGAATTTGCCAATTATGAGTTGGGCGCTTTAACCATTC**
AGCC}{ATGGATGCTTAACAGGAATCATCGTAAATAAAATAACCAAATTCCAATAGACTTATTGAACGTTCCGTTGGCG
TGCATCCAGCAGGAATTGAACCTACGAATTTGCCAATTATGAGTTGGGCGCTTTAACCATTCAGCC}{ATGGATG
CTTAACAGGAATCATCGTAAATAAAATAACCAAATTCCAATAGACTTATTGAACGTTCCGTTGGCG**TGCATCCAGCAG**
GAATTGAACCTACGAATTTGCCAATTATGAGTTGGGCGCTTTAACCATTCAGCC}ATGGATGCTTAACAGGTATCA
TCATAAATAACCAAATTCCAATTTAAATGAAATCTTTAGGAGGAAGCAATAGCA

>*Paris incompleta* – number of tandem repeats: 3 (155 bp). Underlined red letters indicate accumulation of small repeat TA in the third repeat.
AAATCCATTTTCTTCCCTATGAGTTCCAGTATCGATAAGAATTCTAGTTCTTACTGTTCATATGTTATAGTATGAATATA
CCATATCAATTCGTTATGTATGGATGATGAGATTCCATTGATACAGAG{CCAATTCAAATAGACTTATTGAACGTTCCAC
TGGCG**TGCATCCAGCAGGAATTGAACCTACGAATTTGCCAATTATGAGTTGGGCGCTTTAACCATTCAGCCATGG**
ATGCTTAACACTTTACAGGAATGTAAATATAATATATAATATAAATAAC}{CAAATTCCAATAGACTTATTGAACGTTCC
ATTGGCG**TGCATCCAGCAGGAATTGAACCTACGAATTTGCCAATTATGAGTTGGGCGCTTTAACCATTCAGCCAT**
GGATGCTTAACACTTTACAGGAATGTAAATATAATATATAATATAAATAAC}{CAAATTCCAATAGACTTATTGAACGT
TCCATTGGCG**TGCATCCAGCAGGAATTGAACCTACGAATTTGCCAATTATGAGTTGGGCGCTTTAACCATTCAGC**
CATGGATGCTTAACACTTAACAGGAATGTAAATATAATATATAATATATATAAATAAC}CAAATTCCAATTTTAATGAA
ATATTTAGGAGGAAACAATATCA

>*Paris japonica* – Number of repeats: 2 (150 bp) and 2 (116 bp)
AAATCCATTTTCTTCCCTATGAGTTCCAGTATCGATAAGAATTCTAGTTCTTACTGTTCATATGTTATGGTATGAATATA
CCATATCAATTCGTTATGTATGGATGATGAGATTCCATTGATACAGAGCCAATT{CCAATAGACTTATTGAACATTGGCG
TGCATCCAGCAGGAATTGAACCTACGAATTTGCCAATTATGAGTTGGGCGCTTTAACCATTCAGCCATGGATGCT
TAACACTTAACAGGAATCATCGTATATAAATAGAATATAACTAACCAAATT}{CCAATTGACTTATTGAACACAGGAAT
CATCGTATATAAATAGAATATAACTAACCAAATTCGAATTGACTTATTGAACATTGGAATCATCGTAAATAGAATATAA
CTAACGAAATT}CCAATTGACTTATTGAACATTGGAATCATCGTAAATAGAATATAACTAACCAAATT{CCAATTGACTT
ATTGAACACAGGAATCATCGTATATAAATAGAATATAACTAACCAAATTCGAATTGACTTATTGAACATTGGAATCATC
GTAAATAGAATATAACTAACGAAATT}{CCAATAGACTTATTGAACATTGGCG**TGCATCCAGCAGGAATCGAACCTAC**
GAATTTGCCAATTATGAGTTGGGCGCTTTAACCATTCAGCCATGGATGCTTAACACTTAACAGGAATCATCGTATA
TAAATAGAATATAACTAACCAAATT}TGAATTGACTTATTGAACATTGGAATCATCGTAAATAGAATATAACTAACGAA
ATTCCAATTTAAATTCAATCTTTAAGATTTAGGAGGAGACA

>*Paris vietnamensis* – Number of repeats: 2 (29 bp) and 6 (24 bp)
AAATCCATTTTCTTCCCTATGAGTTCCAGTATCGATAAGAATTCTAGTTCTTACTGTTCATATGTTATGGTATGAATATA
CCATATCAATTCGTTATGTATGGATGATGAGATTCCATTGATACAGAGCCAATTCCAATAGACTTATTGAACATTGGCG
TGCATCCAGCAGGAATTGAACCTACGAATTTGCCAATTATGAGTTGGGCGCTTTAACCATTCAGCCATGGATG{C
TTAACAGGAATCATCGTATCGTATATAA}{CTTAACAGGAATCATCGTATATAA}{CTTAACAGGAATCATCGTATATAA}
{CTTAACAGGAATCATCGTATATAA}{CTTAACAGGAATCATCGTATATAA}{CTTAACAGGAATCATCGTATATAA}{CTT
AACAGGAATCATCGTATATAA}{CTTAACAGGAATCATCGTATCGTATATAA}ATATAATATATATATATATAACCAATA
ACCAAATTCCAATTTAAATTAAATCTTTAGGAGGAGGCA

>*Paris dulongensis* – Number of tandem repeats: 16 (24 bp).
AAATCCATTTTCTTCCCTATGAGTTCCAGTATCGATAAGAATTATAGTTCTTACTGTTCATATGTTATGGTATGAATATA
CCATATCAATTCGTTATGTATGGATGATGAGATTCCATTGATACAGAGCCAATTCCAATAGACTTATTGAACATTGGCG
TGCATCCAGCAGGAATTGAACCTACGAATTTGCCAATTATGAGTTGGGCGCTTTAACCATTCAGCCATGGATG{C
TTAACAGGAATCATCGTATATAA}{CTTAACAGGAATCATCGTATATAA}{CTTAACAGGAATCATCGTATATAA}{CTTA
GCAGGAATCATCGTATATAA}{CTTAACAGGAATCATCGTATATAG}{CTTAACAGGAATCATCGTATATAA}{CTTATCA
GGAATCATCGTATATAA}{CTTAACAGGAATCATCGTATACAA}{CTTAACAGGAATCATCGTATATAA}{CTTAACAGGA
ATCATCGTATATAA}{CTTAACAGGAATCATCGTATATAA}{CTTAACAGGAATCATCGTATATAA}{CTTAACAGGAATC
ATCGTATATAA}{CTTAACAGGAATCATCGTATATAA}{CTTAACAGGAATCATCGTATATAA}{CTTAACAGGAATCATC
GTATATAA}CTTAACAGGAATCATCGTATCGTATATAAATATAATATATATATATAACCATAACCAAATATAACCATAA
CCAAATTCCAATTTAAATTAAATCTTTAGGAGGAGGCA

>*Paris axialis* – Number of repeats: 2 (29 p) and 6 (24 bp)
AAATCCATTTTCTTCCCTATGAGTTCCAGTATCGATAAGAATTCTAGTTCTTACTGTTCATATGTTATGGTATGAATATA
CCATATCAATTCGTTATGTATGGATGATGAGATTCCATTGATACAGAGCCAATTCCAATAGACTTATTGAACATTGGCG
TGCATCCAGCAGGAATTGAACCTACGAATTTGCCAATTATGAGTTGGGCGCTTTAACCATTCAGCCATGGATG{C
TTAACAGGAATCATCGTATCGTATATAA}{CTTAACAGGAATCATCGTATATAA}{CTTAACAGGAATCATCGTATATAA}
{CTTAACAGGAATCATCGTATATAA}{CTTAACAGGAATCATCGTATATAA}{CTTAACAGGAATCATCGTATATAA}{CTT
AACAGGAATCATCGTATATAA}{CTTAACAGGAATCATCGTATCGTATATAA}ATATAATATATAACCAACCAAATTCCA
ATTTAAATTAAATCTTTAGGAGGAGGCA

>*Paris thibetica* – Number of tandem repeats: 7 (24 bp)
AAATCCATTTTCTTCCCTATGAGTTCCAGTATCGATAAGAATTCTAGTTCTTACTGTTCATATGTTATGGTATGAATATA
CCATATCAATTCGTTATGTATGGATGATGAGATTCCATTGATACAGAGCCAATTCCAATAGACTTATTGAACATTGGCG
TGCATCCAGCAGGAATTGAACCTACGAATTTGCCAATTATGAGTTGGGCGCTTTAACCATTCAGCCATGGATG{C
TTAACAGGAATCATCGTATATAA}{CTTAACAGGAATCATCGTATATAA}{CTTAACAGGAATCATCGTATATAA}{CTTA
ACAGGAATCATCGTATATAA}{CTTAACAGGAATCATCGTATATAA}{CTTAACAGGAATCATCGTATATAA}{CTTAACA
GGAATCATCGTATATAA}CTTAACAGGAATCATCGTATTCGTATATAAATATAATATATAACCAACCAAATTCCAATTTA
AATTAAATCTTTAGGAGGAGGCA

>*Paris mairei* – Number of tandem repeats: 12 (24 bp)
AAATCCATTTTCTTCCCTATGAGTTCCAGTATCGATAAGAATTATAGTTCTTACTGTTCATATGTTATGGTATGAATATA
CCATATCAATTCGTTATGTATGGATGATGAGATTCCATTGATACAGAGCCAATTCCAATAGACTTATTGAACATTGGCG
TGCATCCAGCAGGAATTGAACCTACGAATTTGCCAATTATGAGTTGGGCGCTTTAACCATTCAGCCATGGATG{C
TTAACAGGAATCATCGTATATAA}{CTTAACAGGAATCATCGTATATAA}{CTTAACAGGAATCATCGTATATAA}{CTTA
ACAGGAATCATCGTATATAA}{CTTAACAGGAATCATCGTATATAA}{CTTAACAGGAATCATCGTATATAA}{CTTAACA
GGAATCATCGTATATAA}{CTTAACAGGAATCATCGTATATAA}{CTTAACAGGAATCATCGTATATAA}{CTTAACAGGA
ATCATCGTATATAA}{CTTAACAGGAATCATCGTATATAA}{CTTAACAGGAATCATCGTATATAA}CTTAACAGGAATCA
TCGTATCGTATATAAATATAATATATATATATAACCATAACCAAATTCCAATTTAAATTAAATCTTTAGGAGGAGGCA

>*Paris polyphylla* var. *polyphylla* – Number of tandem repeats: 8 (24 bp)
AAATCCATTTTCTTCCCTATGAGTTCCAGTATCGATAAGAATTATAGTTCTTACTGTTCATATGTTATGGTATGAATATA
CCATATCAATTCGTTATGTATGGATGATGAGATTCCATTGATACAGAGCCAATTCCAATAGACTTATTGAACATTGGCG
TGCATCCAGCAGGAATTGAACCTACGAATTTGCCAATTATGAGTTGGGCGCTTTAACCATTCAGCCATGGATG{C
TTAACAGGAATCATCGTATATAA}{CTTAACAGGAATCATCGTATATAA}{CTTAACAGGAATCATCGTATATAA}{CTTA
ACAGGAATCATCGTATATAA}{CTTAACAGGAATCATCGTATATAA}{CTTAACAGGAATCATCGTATATAA}{CTTAACA
GGAATCATCGTATATAA}{CTTAACAGGAATCATCGTATATAA}CTTAACAGGAATCATCGTATCGTATATAAATATAAT
ATATATATATAACCATTAACCATAACCAAATTCCAATTTAAATTAAATCTTTAGGAGGAGGCA

>*Paris polyphylla* var. *chinensis* -Number of tandem repeats: 12 (24 bp)
AAATCCATTTTCTTCCCTATGAGTTCCAGTATCGATAAGAATTATAGTTCTTACTGTTCATATGTTATGGTATGAATATA
CCATATCAATTCGTTATGTATGGATGATGAGATTCCATTGATACAGAGCCAATTCCAATAGACTTATTGAACATTGGCG
TGCATCCAGCAGGAATTGAACCTACGAATTTGCCAATTATGAGTTGGGCGCTTTAACCATTCAGCCATGGATG{C
TTAACAGGAATCATCGTATATAA}{CTTAACAGGAATCATCGTATATAA}{CTTAACAGGAATCATCGTATATAA}{CTTA
ACAGGAATCATCGTATATAA}{CTTAACAGGAATCATCGTATATAA}{CTTAACAGGAATCATCGTATATAA}{CTTAACA
GGAATCATCGTATATAA}{CTTAACAGGAATCATCGTATATAA}CTTAACAGGAATCATCGTATCGTATATAAATATAAT
ATATATATATAACCATTAACCATAACCAAATTCCAATTTAAATTAAATCTTTAGGAGGAGGCA

>*Paris polyphylla* var. *stenophylla*- Number of tandem repeats: 13 (24 bp)
AAATCCATTTTCTTCCCTATGAGTTCCAGTATCGATAAGAATTATAGTTCTTACTGTTCATATGTTATGGTATGAATATA
CCATATCAATTCGTTATGTATGGATGATGAGATTCCATTGATACAGAGCCAATTCCAATAGACTTATTGAACATTGGCG
TGCATCCAGCAGGAATTGAACCTACGAATTTGCCAATTATGAGTTGGGCGCTTTAACCATTCAGCCATGGATG{C
TTAACAGGAATCATCGTATATAA}{CTTAACAGGAATCATCGTATATAA}{CTTAACAGGAATCATCGTATATAA}{CTTA
ACAGGAATCATCGTATATAA}{CTTAACAGGAATCATCGTATATAA}{CTTAACAGGAATCATCGTATATAA}{CTTAACA
GGAATCATCGTATATAA}{CTTAACAGGAATCATCGTATATAA}{CTTAACAGGAATCATCGTATATAA}{CTTAACAGGA
ATCATCGTATATAA}{CTTAACAGGAATCATCGTATATAA}{CTTAACAGGAATCATCGTATATAA}{CTTAACAGGAATC
ATCGTATATAA}CTTAACAGGAATCATCGTATCGTATATAAATATAATATATATATATAACCATAACCAAATTCCAATTT
AAATTAAATCTTTAGGAGGAGGCA

>*Paris fargesii* – Number of tandem repeats: 16 (24 bp)
AAATCCATTTTCTTCCCTATGAGTTCCAGTATCGATAAGAATTATAGTTCTTACTGTTCATATGTTATGGTATGAATATA
CCATATCAATTCGTTATGTATGGATGATGAGATTCCATTGATACAGAGCCAATTCCAATAGACTTATTGAACATTGGCG
TGCATCCAGCAGGAATTGAACCTACGAATTTGCCAATTATGAGTTGGGCGCTTTAACCATTCAGCCATGGATG{C
TTAACAGGAATCATCGTATATAA}{CTTAACAGGAATCATCGTATATAA}{CTTAACAGGAATCATCGTATATAA}{CTTA
ACAGGAATCATCGTATATAA}{CTTAACAGGAATCATCGTATATAA}{CTTAACAGGAATCATCGTATATAA}{CTTAACA
GGAATCATCGTATATAA}{CTTAACAGGAATCATCGTATATAA}{CTTAACAGGAATCATCGTATATAA}{CTTAACAGGA
ATCATCGTATATAA}{CTTAACAGGAATCATCGTATATAA}{CTTAACAGGAATCATCGTATATAA}{CTTAACAGGAATC
ATCGTATATAA}{CTTAACAGGAATCATCGTATATAA}{CTTAACAGGAATCATCGTATATAA}{CTTAACAGGAATCATC
GTATATAA}CTTAACAGGAATCATCGTATCGTATATAAATATAATATATATATATAACCATAACCAAATTCCAATTTAAA
TTAAATCTTTAGGAGGAGGCA

>*Paris dunniana* – Number of tandem repeats: 4 (24 bp)
AAATCCATTTTCTTCCCTATGAGTTCCAGTATCGATAAGAATTATAGTTCTTACTGTTCATATGTTATGGTATGAATATA
CCATATCAATTCGTTATGTATGGATGATGAGATTCCATTGATACAGAGCCAATTCCAATAGACTTATTGAACATTGGCG
TGCATCCAGCAGGAATTGAACCTACGAATTTGCCAATTATGAGTTGGGCGCTTTAACCATTCAGCCATGGATG{C
TTAACAGGAATCATCGTATATAA}{CTTAACAGGAATCATCGTATATAA}{CTTAACAGGAATCATCGTATATAA}{CTTA
ACAGGAATCATCGTATATAA}CTTAACAGGAATCATCGTATCGTATATAAATATAATATATATATATAACCATAACCAA
ATTCCAATTTAAATTAAATCTTTAGGAGGAGGCAA

>*Paris luquanensis*- Number of tandem repeats: 13 (24 bp)

AAATCCATTTTCTTCCCTATGAGTTCCAGTATCGATAAGAATTATAGTTCTTACTGTTCATATGTTATGGTATGAATATA
CCATATCAATTCGTTATGTATGGATGATGAGATTCCATTGATACAGAGCCAATTCCAATAGACTTATTGAACATTGGCG
TGCATCCAGCAGGAATTGAACCTACGAATTTGCCAATTATGAGTTGGGCGCTTTAACCATTCAGCCATGGATG{C
TTAACAGGAATCATCGTATATAA}{CTTAACAGGAATCATCGTATATAA}{CTTAACAGGAATCATCGTATATAA}{CTTA
ACAGGAATCATCGTATATAA}{CTTAACAGGAATCATCGTATATAA}{CTTAACAGGAATCATCGTATATAA}{CTTAACA
GGAATCATCGTATATAA}{CTTAACAGGAATCATCGTATATAA}{CTTAACAGGAATCATCGTATATAA}{CTTAACAGGA
ATCATCGTATATAA}{CTTAACAGGAATCATCGTATATAA}{CTTAACAGGAATCATCGTATATAA}{CTTAACAGGAATC
ATCGTATATAA}CTTAACAGGAATCATCGTATCGTATATAAATATAATATATATATATAACCATAACCAAATTCCAATTT
AAATTAAATCTTTAGGAGGAGGCA

>*Paris rugosa*- Number of tandem repeats: 12 (24 bp)

AAATCCATTTTCTTCCCTATGAGTTCCAGTATCGATAAGAATTCTAGTTCTTACTGTTCATATGTTATGGTATGAATATA
CCATATCAATTCGTTATGTATGGATGATGAGATTCCATTGATACAGAGCCAATTCCAATAGACTTATTGAACATTGGCG
TGCATCCAGCAGGAATTGAACCTACGAATTTGCCAATTATGAGTTGGGCGCTTTAACCATTCAGCCATGGATG{C
TTAACAGGAATCATCGTATATAA}{CTTAACAGGAATCATCGTATATAA}{CTTAACAGGAATCATCGTATATAA}{CTTA
ACAGGAATCATCGTATATAA}{CTTAACAGGAATCATCGTATATAA}{CTTAACAGGAATCATCGTATATAA}{CTTAACA
GGAATCATCGTATATAA}{CTTAACAGGAATCATCGTATATAA}{CTTAACAGGAATCATCGTATATAA}{CTTAACAGGA
ATCATCGTATATAA}{CTTAACAGGAATCATCGTATATAA}{CTTAACAGGAATCATCGTATATAA}CTTAACAGGAATCA
TCGTATCGTATATAAATATAATATATATATATAACCATAACCAAATTCCAATTTAAATTAAATCTTTAGGAGGAGGCA

>*Pseudotrillium rivale* – Number of tandem repeats: 0

AAATCCATTTTCTTCCCTATGAGTTCCAGTATCGATAAGAATTCTAGTTCTTACTGTTCATATGTTATGGTATGAATATA
CCATATCAATTCGTTATGTATGGATGATGAAATTCCATTGATACAGAGCCAATTCCAATAGACTTATTGAACGTTCCATT
AGCG**TGCATCCAGCAGGAATTGAACCTACGAATTTGCCAATTATGAGTTGGGCGCTTTAACCATTCAGCCATGGA**
TGCTTAACAGGGATTATCGTAAATAACCAAATTCCAATTGAAATAAAATATTTAGGAGGAATCA

>*Trillium undulatum* - Number of tandem repeats: 0

AAATCCATTTTCTTCCCTATGAGTTCCAGTATCGATAAGAATTCTAGTTCTTACTGTTCATATGTTATGGTATGAATATA
CCATATCAATTCGTTATGTATGGATGATGAGATTCCATTGATACAGAGCCAATTCCAATAGACTTATTGAACGTTCCCAT
TGGCG**TGCATCCAGCAGGAATTGAACCTACGAATTTGCCAATTATGAGTTGGGCGCTTTAACCATTCAGCCATGG**
ATGCTTAACAGGGATCATCGTACATCGTGAATAACCAAATTCCAATTGAAATGAAATCTTTAGGAGGAATCA

>*Trillium tschonoskii* – Number of tandem repeats: 7 (20 bp)
AAATCCATTTTCTTCCCTATGAGTTCCAGTATCGATAAGAATTCTAGTTCTTACTGTTCATATGTTATAGTATGAATATA
CCATATCAATTCGTTATGTATGGATGATGAGATTCCATTGATACAGAGCCAATTCCAATAGACTTATTGAACTGAACGT
TCCATTGGCG**TGCATCCAGCAGGAATTGAACCTACGAATTTGCCAATTATGAGTTGGGCGCTTTAACCATTCAGC**
CATGGATGCTT**{**AACAGGTATTATCATACTGA**}{**AACAGGTATTATCATACTGA**}{**AACAGGTATTATCATACTGA**}{**AAC
AGGTATTATCATACTGA**}{**AACAGGTATTATCATACTGA**}{**AACAGGTATTATCATACTGA**}{**AACAGGTATTATCATACTG
A**}**AACAGGTATTATCATAAATAACCAAATTCCAATTTAAATAAAGGAGGAAGTCATAGTC

>*Trillium smalii*- Number of tandem repeats: 7 (20 bp)
CCATAAATCCATTTTCTTCCCTATGAGTTCCAGTATCGATAAGAATTCTAGTTCTTACTGTTCATATGTTATAGTATGAAT
ATACCATATCAATTCGTTATGTATGGATGATGAGATTCCATTGATACAGAGCCAATTCCAATAGACTTATTGAACTGAA
CGTTCCATTGGCG**TGCATCCAGCAGGAATTGAACCTACGAATTTGCCAATTATGAGTTGGGCGCTTTAACCATTCA**
GCCATGGATGCTT**{**AACAGGTATTATCATACTGA**}{**AACAGGTATTATCATACTGA**}{**AACAGGTATTATCATACTGA**}{**A
ACAGGTATTATCATACTGA**}{**AACAGGTATTATCATACTGA**}{**AACAGGTATTATCATACTGA**}{**AACAGGTATTATCATA
CTGA**}**AACAGGTATTATCATAAATAACCAAATTCCAATTTAAATAAAGGAGGAAGTCATAGTC

>*Trillium flexipes*- Number of repeats: 5 (18 bp) and 13 (20 bp)

CCATAAATCCATTTTCTTCCCTATGAGTTCCAGTATCGATAAGAATTCTAGTTCTTACTGTTCATATGTTATAGTATGAATATACCATATCAATTCGTTATGTATGGATGATGAGATTCCATTGATACAGAGCCAATTCCAATAGACTTATTGAACTGAACGTTCCATTGGCG**TGCATCCAGCAGGAATTGAACCTACGAATTTGCCAATTATGAGTTGGGCGCTTTAACCATTCAGCCATGGATG**CTT{AACAGGTATTATCATACTGA}{AACAGGTATTATCATACTGA}{AACATATTATCATACTGA}{AACAGGTATTATCATACTGA}{AACAGGTATTATCATACTGA}{AACAGGTATTATCATACTGA}{AACATATTATCATACTGA}{AACAGGTATTATCATACTGA}{AACAGGTATTATCATACTGA}{AACAGGTATTATCATACTGA}{AACATATTATCATACTGA}{AACAGGTATTATCATACTGA}{AACATATTATCATACTGA}{AACAGGTATTATCATACTGA}{AACATATTATCATACTGA}{AACAGGTATTATCATACTGA}{AACAGGTATTATCATACTGA}{AACAGGTATTATCATACTGA}AACATATTCCAATTTAAATAAAGGAGGAAGTAATAGTA

>*Trillium rugelii*- Number of repeats: 9 (18bp) and 11 (20 bp)

AAATCCATTTTCTTCCCTATGAGTTCCAGTATCGATAAGAATTCTAGTTCTTACTGTTCATATGTTATAGTATGAATATACCATATCAATTCGTTATGTATGGATGATGAGATTCCATTGATACAGAGCCAATTCCAATAGACTTATTGAACTGAACGTTCCATTGGCG**TGCATCCAGCAGGAATTGAACCTACGAATTTGCCAATTATGAGTTGGGCGCTTTAACCATTCAGCCATGGATG**CTT{AACAGGTATTATCATACTGA}{AACAGGTATTATCATACTGA}{AACATATTATCATACTGA}{AACAGGTATTATCATACTGA}{AACATATTATCATACTGA}{AACATATTATCATACTGA}{AACAGGTATTATCATACTGA}{AACATATTATCATACTGA}{AACAGGTATTATCATACTGA}{AACATATTATCATACTGA}{AACAGGTATTATCATACTGA}{AACATATTATCATACTGA}{AACATATTATCATACTGA}{AACAGGTATTATCATACTGA}{AACATATTATCATACTGA}{AACAGGTATTATCATACTGA}{AACATATTATCATACTGA}{AACAGGTATTATCATACTGA}{AACAGGTATTATCATACTGA}{AACAGGTATTATCATACTGA}AACATATTCCAATTTAAATAAAGGAGGAAATAATAGTA

>*Trillium erectum*- Number of repeats: 9 (18bp) and 11 (20 bp)

CCATAAATCCATTTTCTTCCCTATGAGTTCCAGTATCGATAAGAATTCTAGTTCTTACTGTTCATATGTTATAGTATGAAT
ATACCATATCAATTCGTTATGTATGGATGATGAGATTCCATTGATACAGAGCCAATTCCAATAGACTTATTGAACTGAA
CGTTCCATTGGCG**TGCATCCAGCAGGAATTGAACCTACGAATTTGCCAATTATGAGTTGGGTGCTTTAACCATTCA**
GCCATGGATGCTT{AACAGGTATTATCATACTGA}{AACAGGTATTATCATACTGA}{AACATATTATCATACTGA}{AAC
AGGTATTATCATACTGA}{AACATATTATCATACTGA}{AACATATTATCATACTGA}{AACAGGTATTATCATACTGA}{A
ACATATTATCATACTGA}{AACAGGTATTATCATACTGA}{AACATATTATCATACTGA}{AACAGGTATTATCATACTGA}{
AACATATTATCATACTGA}{AACATATTATCATACTGA}{AACAGGTATTATCATACTGA}{AACATATTATCATACTGA}{A
ACAGGTATTATCATACTGA}{AACATATTATCATACTGA}{AACAGGTATTATCATACTGA}{AACAGGTATTATCATACTG
A}{AACAGGTATTATCATACTGA}AACATATTCCAATTTAAATAAAGGAGGAAATAATAGTA

>*Trillium simile*- Number of repeats: 8 (18 bp) and 11 (20 bp)

CCATAAATCCATTTTCTTCCCTATGAGTTCCAGTATCGATAAGAATTCTAGTTCTTACTGTTCATATGTTATAGTATGAAT
ATACCATATCAATTCGTTATGTATGGATGATGAGATTCCATTGATACAGAGCCAATTCCAATAGACTTATTGAACTGAA
CGTTCCATTGGCG**TGCATCCAGCAGGAATTGAACCTACGAATTTGCCAATTATGAGTTGGGCGCTTTAACCATTCA**
TCCATGGATGCTT{AACAGGTATTATCATACTGA}{AACAGGTATTATCATACTGA}{AACATATTATCATACTGA}{AAC
AGGTATTATCATACTGA}{AACATATTATCATACTGA}{AACATATTATCATACTGA}{AACATATTATCATACTGA}{AACA
GGTATTATCATACTGA}{AACATATTATCATACTGA}{AACAGGTATTATCATACTGA}{AACATATTATCATACTGA}{AAC
AGGTATTATCATACTGA}{AACATATTATCATACTGA}{AACAGGTATTATCATACTGA}{AACAGGTATTATCATACTGA}
{AACATATTATCATACTGA}{AACAGGTATTATCATACTGA}{AACAGGTATTATCATACTGA}{AACAGGTATTATCATAC
TGA}AACATATTCCAATTTAAATAAAGGAGGAAGTAATAGTA

>*Trillium decumbens*- Number of tandem repeats: 0

AAATCCATTTTCTTCCCTATGAGTTCCAGTATCGATAAGAATTCTAGTTCTTACTGTTCATATGTTATGGTATGAATATA
CTCTATCAATTCGTTATGTATGGATGATGAGATTCCATTGATACAGAGCCAATTCCAATAGACTTGACTTATGAAACAG
TTTCAAATAGAATTGAGAATTGACTTATGAGACGGTTCCACTGGTG**TGCATCCAGTAGGAATCGAACCTACGGATTT**
GCCAATTATGAGTTGGGCGCTTTAACCATTCAGCCATGGATGCTTAACTTCACAGGGATTCTCAATTATCCTAAATA
ATATATAATATTATAATATCATAATATCATAAACAACCCAATTCCAATTTTTATCAAATCTTTATCTTTATTAGGAGGAA
GTAATAGTA

>*Trillium cuneatum*- Number of tandem repeats: 0

AAATCCATTTTCTTCCCTATGAGTTCCAGTATCGATAAGAATTCTAGTTCTTACTGTTCATATGTTATGGTACGAATATA
CTATACCCTATCAATTCGTTATGTATGGATGATGAGATTCCATTGATACAGAGCCAATTCCAATAGACTTGACTTATGA
AACAGTTTCCAATAGAATTGAGAATTGACTTATGAGACGGTTCCATTGGTG**TGCATCCAGTAGGAATCGAACCTACG**
GATTTGCCAATTATGAGTTGGGCGCTTTAACCATTCAGCCATGGATGCTTAACAGGGATTCTCAATTATCATCAAT
AATATCATAACATAAATAACCCAATTCCAATTTTCTTCATCAAATCTTTAGGAGGAAGTAATAGTA

> *Trillium underwoodii*- Number of repeats: 2 (155 bp) and 2 (33 bp)

AAATCCATTTTCTTCCCTATGAGTTCCAGTATCGATAAGAATTCTAGTTCTTACTGTTCATATGTTATGGTATGAATATA
CCCTATCAATTCGTTATGTATGGATGATGAGATTCCATTGATACAGAGC{CAATTCCAATAGACTTGACTTATGAAACAG
TTTCCAATAGAATTGAGAATTGACTTATGAGACGGTTCCATTGGTG**TGCATCCAGTAGGAATCGAACCTACGGATTT**
GCCAATTAAGAGTTGGGCGCTTTAACCATTCAGCCATGGATGCTTAAC}{AGGGATTCTCAATTATCCTAAATAATA
TCATAA}CTATAACATAAATAACCAAATT{CAATTCCAATAGACTTGACTTATGAAACAGTTTCCAATAGAATTGAGAAT
TGACTTATGAGACGGTTCCATTGGTG**TGCATCCAGTAGGAATCGAACCTACGGATTTGCCAATTAAGAGTTGGGCG**
CTTTAACCATTCAGCCATGGATGCTTAAC}TTAACCTTAAC{AGGGATTCTCAATTATCCTAAATAATATCATAA}TAT
AACTATAACATAAATAACCCAATTCCAATTTTTATCAAATCTTTAGGAGGAAGTAATAGTA

>*Trillium chloropetalum* - Number of tandem repeats: 2 (209 bp)
AAATCCATTTTCTTCCCTATGAGTTCCAGTATCGATAAGAATTCTAGTTCTTACTGTTCATATGTTATGGTACGAATATA
CCCTATCAATTCGTTATGTATGGATGATGAGATTCCATTGATACAGAG{CCAATTCCAATAGACTTGACTTATGAAACAG
TTTCCAATAGAATTGAGAATTGACTTATGAGACGGTTCCATTGGTG**TGCATCCAGTAGGAATCGAACCTACGGATTT**
GCCAATTATGAGTTGGGCGCTTTAACCATTCAGCCATGGATGCTTAACAGGGATTCTCAATTATCCATCAATAATAT
CATAACATAAATAAC}{CCAATTCCAATAGACTTGACTTATGAAACAGTTTCCAATAGAATTGAGAATTGACTTATGAGA
CGGTTCCATTGGTG**TGCATCCAGTAGGAATCGAACCTACGGATTTGCCAATTATGAGTTGGGCGCTTTAACCATTC**
AGCCATGGATGCTTAACAGGGATTCTCAATTATCATCAATAATATCATAACATAAATAAC}CCAATTCCAATTTTCTTC
ATCAAATCTTTAAATCTTTAGGAGGAAGTAATAGTA

>*Trillium luteum* - Number of repeats: 2 (181 bp)
AAATCCATTTTCTTCCCTATGAGTTCCAGTATCGATAAGAATTCTAGTTCTTACTGCTCATATGTTATGGTACGAATATA
CTATACCCTATCAATTCGTTATGTATGGATGATGAGATTCCATTGATACAGAG{CCAATTCCAATAGACTTGACTTATGA
AACAGTTTCCAATAGAATTGAGAATTGACTTATGAGACGGTTCCATTGGTG**TGCATCCAGTAGGAATCGAACCTACG**
GATTTGCCAATTATGAGTTGGGCGCTTTAACCATTCAGCCATGGATGCTTAACAGGGATTCTCAATTATCATCAAT
AA}CCAAATT{CAATTCCAATAGACTTGACTTATGAAACAGTTTCCAATAGAATTGAGAATTGACTTATGAGACGGTTCC
ATTGGTG**TGCATCCAGTAGGAATCGAACCTACGGATTTGCCAATTATGAGTTGGGCGCTTTAACCATTCAGCCAT**
GGATGCTTAACAGGGATTCTCAATTATCATCAATAA}TATCATAACATAAATAACCCAATTCCAATTTTCTTCATCAAAT
CTTTAGGAGGAAGTAATAGTA

>*Trillium sessile* - Number of repeats: 2 (181 bp)
AAATCCATTTTCTTCCCTATGAGTTCCAGTATCGATAAGAATTCTAGTTCTTACTGTTCATATGTTATGGTACGAATATA
CTATACCCTATCAATTCGTTATGTATGGATGATGAGATTCCATTGATACAGAG{CCAATTCCAATAGACTTGACTTATGA
AACAGTTTCCAATAGAATTGAGAATTGACTTATGAGACGGTTCCATTGGTG**TGCATCCAGTAGGAATCGAACCTACG**
GATTTGCCAATTATGAGTTGGGCGCTTTAACCATTCAGCCATGGATGCCTTAACAGGGATTCTCAATTATCATCAAT
AA}CCAAATT{CAATTCCAATAGACTTGACTTATGAAACAGTTTCCAATAGAATTGAGAATTGACTTATGAGACGGTTCC
ATTGGTG**TGCATCCAGTAGGAATCGAACCTACGGATTTGCCAATTATGAGTTGGGCGCTTTAACCATTCAGCCAT**
GGATGCTTAACAGGGATTCTCAATTATCATCAATAA}TATCATAACATAAATAACCCAATTCCAATTTTCTTCATCAAAT
CTTTAGGAGGAAGTAATAGTA

>*Trillium maculatum*- Number of repeats: 2 (181 bp)

CCATAAATCCATTTTCTTCCCTATGAGTTCCAGTATCGATAAGAATTCTAGTTCTTACTGTTCATATGTTATGGTACGAA
TATACTATACCCTATCAATTCGTTATGTATGGATGATGAGATTCCATTGATACAGAG{CCAATTCCAATAGACTTGACTT
ATGAAACAGTTTCCAATAGAATTGAGAATTGACTTATGAGACGGTTCCATTGGTG**TGCATCCAGTAGGAATCGAACC**
TACGGATTTGCCAATTATGAGTTGGGCGCTTTAACCATTCAGCCATGGATGCTTAACAGGGATTCTCAATTATCAT
CAATAA}CCAAATT{CAATTCCAATAGACTTGACTTATGAAACAGTTTCCAATAGAATTGAGAATTGACTTATGAGACGG
TTCCATTGGTG**TGCATCCAGTAGGAATCGAACCTACGGATTTGCCAATTATGAGTTGGGCGCTTTAACCATTCAG**
CCATGGATGCTTAACAGGGATTCTCAATTATCATCAATAA}TATCATAACATAAATAACCCAATTCCAATTTTCTTCAT
CAAATCTTTAGGAGGAAGTAATAGTA

>*Trillium govanianum*- Number of tandem repeats: 3 (154 bp)

AAATCCATTTTCTTCCCTATGAGTTCCAGTATCGATAAGAATTCTAGTTCTTACTGTTCATATGTTATGGTATGAATATA
CCATATCAATTCGTTATGTATGGATGATGAG{ATTCCATTGATACAGAGCCAATTCCAATAGACTTATTGAACGTTCCAT
TGGCG**TGCATCCAGCAGGAATTGAACCTACGAATTTGCCAATTATGAGTTGGGCGCTTTAACCATTCAGCCATGG**
ATGCTTAACAGGGATTATCGTAAATAACCAA}{ATTCCATTGATACAGAGCCAATTCCAATAGACTTATTGAACGTTCCA
TTAGCG**TGCATCCAGCAGGAATTGAACCTACGAATTTGCCAATTATGAGTTGGGCGCTTTAACCATTCAGCCATG**
GATGCTTAACAGGGATTATCGTAAATAACCAA}{ATTCCAATTGATACAGAGCCAATTCCAATAGACTTATTGAACGTT
CCATTAGCG**TGCATCCAGCAGGAATTGAACCTACGAATTTGCCAATTATGAGTTGGGCGCTTTAACCATTCAGCC**
ATGGATGCTTAACAGGGATTATCGTAAATAACCAA}ATTCCAATTGAAATAAAATATTTAGGAGGAAACAAACA

>*Xerophyllun tenax*- Number of tandem repeats: 0

AAATCCATTTTCTTCCCTATGAGTTCCAGTATCGATAAGAATTCTAGTTCTTACTGTTCATATGTTATGGTATGAATATA
CCATACCAATTCGTTATGTATGGATGATGAGATTCCATTGATACAGAGCCAATTCCAATAGACTTATTGAACGTTCCATT
GGCG**TGCATCCAGCAGGAATTGAACCTACGAATTTGCCAATTATGAGTTGGGCGCTTTAACCATTCAGCCATGGA**
TGCTTAACAGGGATCATCGTACATCGTGAATAACCAAATTCCAATTGAAATGAAATCTTTAGGAGGAATCA

>*Xerophyllun asphodeloides*- Number of tandem repeats: 0
AAATCCATTTTCTTCCCTATGAGTTCCAGTATCGATAAGAATTCTAGTTCTTACTGTTCATATGTTATGGTATGAATATA
CCATACCAATTCGTTATGTATGGATGATGAGATTCCATTGATACAGAGCCAATTCCAATAGACTTATTGAACGTTCCATT
GGCG**TGCATCCAGCAGGAATTGAACCTACGAATTTGCCAATTATGAGTTGGGCGCTTTAACCATTCAGCCATGGA**
TGCTTAACAGGGATCATCGTACATCGTGAATAACCAAATTCCAATTGAAATGAAATCTTTAGGAGGAATCA

>*Chionographis japonica*-Number of tandem repeats: 0
AAATCCATTTTCTTCCCTATGAGTTCCAGTATCGATAAGAATTCTAGTTCTTACTGTTCATATGTTATGGTATGAATATA
CCAATTCGTTATGTATGTATGATGAGATTCCATTGATACAGAGCCAATTCCAATAGACTTATTGAACGTTCCCATTGGCG
TGCATCCAGCAGGAATTGAACCTACGAATTTGCCAATTATGAGTTGGGCGCTTTAACCATTCAGCCATGGATGCT
TAACAAGGATCATCGTACATCGTGAATAACCAAATTCCAATTGAAATGAAATCTTTAGGAGGAATCA

>*Heloniopsis tubiflora*- Number of tandem repeats: 0
AAATCCATTTTCTTCCCTATGAGTTCCAGTATCGATAAGAATTCTAGTTCTTACTGTTCATATGTTATGGTATGAATATA
CCAATTCGTTATGTATGGATGATGAGATTCCATTGATACAGAGCCAATTCCAATAGACTTATTGAACGTTCCCATTGGC
G**TGCATCCAGCAGGAATTGAACCTACGAATTTGCCAATTATGAGTTGGGCGCTTTAACCATTCAGCCATGGATG**
CTTAACAGGGATCATCGTACATCGTGAATAACCAAATTCCAATTGAAATGAAATCTTTAGGAGGAATCA

>*Veratrum patulum*- Number of tandem repeats: 0
TTTCTTCCCTATGAGTTCCAGTATCGATAAGAATTAGAGTTCTTACTGTTCATATGTTATGGTATGAATATACCATACCA
ATTCATTATGTATGGATGATGAGATTCCATTGATACAGAGCCAATTCCAATAGACTTATTGGACGTTCCCATTGGCG**TG**
CATCCAGCAGGAATTGAACCTACGAATTTGCCAATTATGAGTTGGGCGCTTTAACCATTCAGCCATGGATGCTAA
ACAGGGATCATCGTACATCGTGAATAAAATAACTAAATTCCAATTGAAATGAAATCGTTAGGAGAAATCA

> *Toxicoscordion micranthus* - Number of tandem repeats: 0
TTTCTTCCCTATGAGTTCCAGTATCGATAATAATTAGAGTTCTTACTGTTCATATGTTATGGTATGAATATACCATACCA
ATTCATTATGTATGGATGATGAGATTCCATTGATACAGAGCCAATTCCAATAGACTTATTGGACGTTCCCATTGGCG**TG**
CATCCAGCAGGAATTGAACCTACGAATTTGCCAATTATGAGTTGGGCGCTTTAACCATTCAGCCATGGATGCTAA
ACAGGGATCATCGTACATCGTGAATAAAATAACCAAATTCCAATTGAAATGAAATCGTTAGGAGAAATCA

> *Anticlea elegans* - Number of repeats: 2 (19 bp)
TTTCTTCCCTATGAGTTCCAGTATCGATAAGAATTCTAGTTCTTACTGTTCATATGTTATGGTATGAATATACCATACCA
ATTCGTTATGTATGGATGATGAGATTCCATTGATACAAAGCCAATTCCAATAGACTTATTGAACGTTCCCATTGGCG**TG**
CATCCAGCAGGAATTGAACCTACGAATTTGCCAATTATGAGTTGGGCGCTTTAACCATTCAGCCATGGATGCTTA
ACAGGGCTCATCGTACATCGTGAATAACCAAATTCCAATTGAAATG{AAATCTTTAGGAGGAATCA}ATGA{AAATCTTT
AGGAGGAATCA}

> *Stenanthium densum* - Number of repeats: 2 (19 bp)
TTTCTTCCCTATGAGTTCCAGTATCGATAAGAATTCTAGTTCTTACTGTTCATATGTTATGGTATGAATATACCATACCA
ATTCGTTATGTATGGATGATGAGATTCCATTGATACAAAGCCAATTCCAATAGACTTATTGAACGTTCCCATTGGCG**TG**
CATCCAGCAGGAATTGAACCTACGAATTTGCCAATTATGAGTTGGGCGCTTTAACCATTCAGCCATGGATGCTTA
ACAGGGCTCATCGTACATCGTGAATAACCAAATTCCAATTGAAATG{AAATCTTTAGGAGGAATCA}ATGA{AAATCTTT
AGGAGGAATCA}

> *Schoenocaulon coricifolium* - Number of tandem repeats: 0
TTTCTTCCCTATGAGTTCCAGTATCGATAAGAATTAGAGTTCTTACTGTTCATATGTTATGGTATGAATATACCATACCA
ATTCATTATGTATGGATGATGAGATTCCATTGATACAGAGCCAATTCCAATAGACTTATTGGACGTTCCCATTGGCG**TG**
CATCCAGCAGGAATTGAACCTACGAATTTGCCAATTATGAGTTGGGCGCTTTAACCATTCAGCCATGGATGCTAA
ACAGGGATCATCGTACATCGTGAATAAAATAACCAAATTCCAATTGAAATGAAATCGTTAGGAGAAATCA

> *Zigadenus glaberrimus* - Number of tandem repeats: 0
TTTCTTCCCTATGAGTTCCAGTATCGATAAGAATTCTAGTTCTTACTGTTCATATGTTATGGTATGAATATACCATACCA
ATTCGTTATGTATGGATGATGAGATTCCATTGATACAGAGCCAATTCCAATAGACTTATTGAACGTTCCCATTGGCG**TG**
CATCCAGCAGGAATTGAACCTACGAATTTGCCAATTATGAGTTGGGCGCTTTAACCATTCAGCCATGGATGCTTA
ACAGGGATCATCGTACATCGTGAATAACCAAATTCCAATTGAAATGAAATCTTTAGGAGGAATCA

>*Lilium longiflorum*- Number of tandem repeats: 0
AAATCCATTTTCTTCCCTATGAGTTCCAGTATCAATAAGAATTCTAGTTCTTACTGTTCATATGTTATGGTATGAATATA
CCATACCAATTCGGTATGTATGGATGATGAGATTCCATTGATACAGAGCCAATTCTAATAGACTTATTGAACGTTCCCA
TTGGCG**TGCATCCAGCAGGAATTGAACCTACGAATTTGCCAATTATGAGTTGGGCGCTTTAACCATTCAGCCATG**
GATGCTTAACAGGGATCATCGTACATCGTAAATAACCAAATTCCAATTGAAATGAAATCTTTAGGAGGAATCA

>*Fritillaria cirrhosa*- Number of tandem repeats: 0
AAATCCATTTTCTTCCCTATGAGTTCCAGTATCAATAAGAATTCTAGTTCTTACTGTTCATATGTTATGGTATGAATATA
CCATACCAATTCGGTATGTATGGATGATGAGATTCCATTGATACAGAGCCAATTCTAATAGACTTATTGAACGTTCCCA
TTGGCG**TGCATCCAGCAGGAATTGAACCTACGAATTTGCCAATTATGAGTTGGGCGCTTTAACCATTCAGCCATG**
GATGCTTAACAGGGATCATCGTACATCGTAAATAACCAAATTCCAATTGAAATGAAATCTTTAGGAGGAATCA

>*Calochortus venustus*- Number of tandem repeats: 0
AAATCCATTTTCTTCCCTATGAGTTCCAGTATCGATAAGAATTCGAGTTCTTACTGTTCATATGTTATGGTATGAATATA
CCATACCAATTCGTTATGTATGGATGATGAGATTCCATTGATACAGAGCCAATTCCAATAGACTTATTGAACGTTCCCA
TTGGCG**TGCATCCAGCAGGAATTGAACCTACGAATTTGCCAATTATGAGTTGGGCGCTTTAACCATTCAGCCATG**
GATGCTTAACAGGGATCATCGTACATCGTGAATAACCAAATTCCAATTGAAATGAAATCTTTAGGAGGAATCA

>*Tricyrtis macropoda*- Number of tandem repeats: 0
AAATCCATTTTATTCCCTATGAGTTCCAGTATCGATAAGAATTCTAGTTCTTACTGTTCATATGTTATGGTATGAATATA
CCATACCAATTCGTTATGTATGGATGATGAGATTCCATTGATACAGAGCCAATTCCAATAGACTTATTGAACGTTCCCA
TTGGCG**TGCATCCAGCAGGAATTGAACCTACGAATTTGCCAATTATGAGTTGGGCGCTTTAACCATTCAGCCATG**
GATGCTTAACAGGTATCATCGTACATCGTGAATAACCAAATTCCAATTTAAATGAAATCTTTAGGAGGAATCA

>*Gagea triflora*- Number of tandem repeats: 0
AAATCCATTTTCTTCCCTATGAGTTCCAGTATCGATAAGAATTCTAGTTCTTACTGTTCATATGTTATGGTATGAATATA
CCATACCAATTCGCTATGTATGGATGACGAGATTCCATTGATACAGAGCCAATTCTAATAGACTTATTGAACGTTCCCA
TTGGCG**TGCATCCAGCAGGAATTGAACCTACGAATTTGCCAATTATGAGTTGGGCGCTTTAACCATTCAGCCATG**
GATGCTTAACAGGGATCATCGTACATCGTGAATAACCAAATTCCAATTGAAATGAAATCTTTAGGAGGAATCA

>*Erythronium japonicum*- Number of tandem repeats: 0
AAATCCATTTTCTTCCCTATGAGTTCCAGTATCGATAAGAATTCTAGTTCTTACTGTTCATATGTTATGGTATGAATATA
CCATACCAATTCGGTATGTATGGATGATGAGATTCCATTGATACAGAGCCAATTCTAATAGACTTATTGAACGTTCCCA
TTGGCG**TGCATCCAGCAGGAATTGAACCTACGAATTTGCCAATTATGAGTTGGGCGCTTTAACCATTCAGCCATG**
GATGCTTAACAGGGATCATCGTACATCGTGAATAACCAAATTCCAATTGAAATGAAATCTTTAGGAGGAATCA

>*Tulipa sylvestris*- Number of tandem repeats: 0
AAATCCATTTTCTTCCCTATGAGTTCCAGTATCGATAAGAATTCTAGTTCTTACTGTTCATATGTTATGGTATGAATATA
CCATACCAATTCGGTATGTATGGATGATGAGATTCCATTGATACAGAGCCAATTCTAATAGACTTATTGAACGTTCCCA
TTGGCG**TGCATCCAGCAGGAATTGAACCTACGAATTTGCCAATTATGAGTTGGGCGCTTTAACCATTCAGCCATG**
GATGCTTAACAGGGATCATCGTACATCGTGAATAACCAAATTCCAATTGAAATGAAATCTTTAGGAGGAATCA

>*Clintonia udensis*- Number of tandem repeats: 0
AAATCCATTTTCTTCCCTATGAGTTCCAGTATCGATAAGAATTCTAGTTCTTACTGTTCATATGTTATGGTATGAATATA
CCATACCAATTCGCTATGTATGGATGATGAGATTCCATTGATACAGAGCCAATTCTAATAGACTTATTGAACGTTCCCA
TTGGCG**TGCATCCAGCAGGAATTGAACCTACGAATTTGCCAATTATGAGTTGGGCGCTTTAACCATTCAGCCATG**
GATGCTTAACAGGGATCATCGTACATCGTGAATAACCAAATTCCAATTGAAATGAAATCTTTAGGAGGAATCA

>*Streptopus ovalis*- Number of tandem repeats: 0
AAATCCATTTTCTTCCCTATGAGTTCCAGTATCGATAAGAATTCTAGTTCTTACTGTTCATATGTTATGGTATGAATATA
CCATACCAATTCGTTATGTATGGATGATGAGATTCCATTGATACAGAGCCAATTCCAATAGACTTATTGAACGTTCCCA
TTGGCG**TGCATCCAGCAGGAATTGAACCTACGAATTTGCCAATTATGAGTTGGGCGCTTTAACCATTCAGCCATG**
GATGCTTAACAGGGATCATCGTACATCGTAAATAACCAAATTCCAATTGAAATGAAATCTTTAGGAGGAATCA

>*Smilax china*- Number of tandem repeats: 0
AAATCCATTTTATTCCCTATGAGTTCCAGTATCGATAAGAATTCTAGTTCTTACTGTTCATATGTTATGGTATGAATATA
CCATACCAATTCGTTATGTATGGATGATGAGATTCCATTGATACAGAGCCAATTCCAATAGACTTATTGAACGTTCCCA
TTGGCG**TGCATCCAGCAGGAATTGAACCTACGAATTTGCCAATTATGAGTTGGGCGCTTTAACCATTCAGCCATG**
GATGCTTAACAGGGATCATCGTACATCGTGAATAACCAAATTCCAATTGAAATGAAATCTTTAGGAGGAGTCA

>*Smilax nipponica*- Number of tandem repeats: 0
AAATCCATTTTCTTCCCTATGAGTTCCAGTATCGATAAGAATTCTAGTTCTTACTGTTCATATGTTATGGTATGAATATA
CCATACCAATTCGTTATGTATGGATGATGAGATTCCATTGATACAGAGCCAATTCCAATAGACTTATTGAACGTTCCCA
TTGGCG**TGCATCCAGCAGGAATTGAACCTACGAATTTGCCAATTATGAGTTGGGCGCTTTAACCATTCAGCCATG**
GATGCTTAACAGGGATCATCGTACATCGTGAATAACCAAATTCCAATTGAAATGAAATCTTTAGGAGGAGTCA

>*Smilax glyciphylla*- Number of tandem repeats: 0
AAATCCATTTTCTTCCCTATGAGTTCCAGTATCGATAAGAATTCTAGTTCTTACTGTTCATATGTTATGGTATGAATATA
CCATACCAATTCTTTATGTATGGATGATGAGATTCCATTGATACAGAGCCAATTCCAATAGACTTATTGAACGTTCCCAT
TGGCG**TGCATCCAGCAGGAATTGAACCTACGAATTTGCCAATTATGAGTTGGGCGCTTTAACCATTCAGCCATGG**
ATGCCTTAACAGGGATCATCGTACATCGTGAATAACCAAATTCCAATTGAAATGAAATCTTTAGGAGGAGTCA

>*Heterosmilax china*- Number of tandem repeats: 0
AAATCCATTTTCTTCCCTATGAGTTCCAGTATCGATAAGAATTCTAGTTCTTACTGTTCATATGTTATGGTATGAATATA
CCATACCAATTCGTTATGTATGGATGATGAGATTCCATTGATACAGAGCCAATTCCAATAGACTTATTGAACGTTCCCA
TTGGCG**TGCATCCAGCAGGAATTGAACCTACGAATTTGCCAATTATGAGTTGGGCGCTTTAACCATTCAGCCATG**
GATGCCTTAACAGGGATCATCGTACATCGTGAATAACCAAATTCCAATTGAAATGAAATCTTTAGGAGGAGTCA

>*Philesia magellanica*- Number of tandem repeats: 0
AAATCCATTTTCTTCCCTATGAGTTCCAGTATCGATAAGAATTCTAGTTCTTACTGTTCATATGTTATGGTATGAATATA
CCATACCAATTCGTTATGTATGGATGATGAGATTCCATTGATACAGAGCCAATTCCAATAGACTTATTGAACGTTCCCA
TTGGCGTGCA**TCCAGCAGGAATTGAACCTACGAATTTGCCAATTATGAGTTGGGCGCTTTAACCATTCAGCCATG**
GATGCCTTAACAGGGATCATCGTACATCGTGAATAACCAAATTCCAATTGAAATGAAATCTTTAGGAGGAATCA

>*Rhipogonum scandens*- Number of tandem repeats: 0
AAATCCATTTTCTTCCCTATGAGTTCCAGTATCGATAAGAATTCTAGTTCTTACTGTTCATATGTTATGGTATGAATATA
CCATACCAATTCGTTATGTATGGATGATGAGATTCCATTGATACAGAGCCAATTCCAATAGACTTATTGAACGTTCCCA
TTGGCG**TGCATCCAGCAGGAATTGAACCTACGAATTTGCCAATTATGAGTTGGGCGCTTTAACCATTCAGCCATG**
GATGCCTTAACAGGGATCATCGTACATCGTGAATAACCAAATTCCAATTGAAATGAAATCTTTAGGAGGAATCA

>*Colchicum autumnale*- Number of tandem repeats: 0
AAATCCATTTTCTTCCCTATGAGTTCCAGTATCGATAAGAATTCTAGTTCTTACTGTTCATATGTTATGGTATGAATATA
CCATACCAATTCGTTATGTATGGATGATGAGATTCCATTGATACAGAGCCAATTACAATAGACTTATTGGACGTTCCCA
TTGGCG**TGCATCCAGCAGGAATTGAACCTACGAATTTGCCAATTATGAGTTGGGCGCTTTAACCATTCAGCCATG**
GATGCCTTAACAGGGATTCGCGAATAACCAAATTTCAATTGAAATGAAATCCTTAGGAGGAATCA

>*Gloriosa superba*- Number of tandem repeats: 0
AAATCCATTTTCTTCCCTATGAGTTCCAGTATCGATAAGAATTCTAGTTCTTACTGTTCATATGTTATGGTATGAATATA
CCATACCAATTCGTTATGTATGGATGATGAGATTCCATTGATACAGAGTCAATTCCAATAGACTTATTGAACGTTCCCAT
TGGCG**TGCATCCAGCAGGAATTGAACCTACGAATTTGCCAATTATGAGTTGGGCGCTTTAACCATTCAGCCATGG**
ATGCCTTAACGGGGAATATCGTACATCGTGAATAACCAAATTCCAATTGAAATGAAATCTTTAGGAGGAATCA

>*Wurmbea burtii*- Number of tandem repeats: 0
AAATCCATTTTCTTCCCTATGAGTTCCAGTATCGATAAGAATTCTAGTTCTTACTGTTCATATGTTATGGTATGAATATA
CCATACCAATTCGTTATGTATGGATGATGAGATTCCATTGGAGCCAAATACAATCGACTTATTGAACGTTCCCATTGGC
G**TGCATCCAGCAGGAATTGAACCTACGAATTTGCCAATTATGAGTTGGGCGCTTTAACCATTCAGCCATGGATG**
CTTATCCGGGATTATCGTACATCGTGAATAACCAAATTCCAATTGAAATGAAACCTTTAGGAGGAATCA

>*Tripladenia cunninghamii*- Number of tandem repeats: 0
TTTCTTCCCTATGAGTTCCAGTATCGATAAGAATTCTAGTTCTTACTGTTCATATGTTATGGTATGAATATACCATACCA
ATTCGTTATGTATGGATGATGAGATTCCATTGATACAGAGCCAATTCCAATAGACTTATTGAACGTTCCCATTGGCG**TG**
CATCCAGCAGGAATTGAACCTACGAATTTGCCAATTATGAGTTGGGCGCTTTAACCATTCAGCCATGGATGCTTA
ACAGGGATTATCGTACATCGTGAATAACCAAATTCCAATTGAAATGAAATCTTTAGGAGGAATCA

>*Uvularia grandiflora*- Number of tandem repeats: 0
AAATCCATTTTCTTCCCTATGAGTTCCAGTATCGATAAGAATTCTAGTTCTTACTGTTCATATGTTATGGTATGAATATA
CCATACCAATTCGTTATGTATGGATGATGAGATTCCATTGATACAGAGCCAATTCCAATAGACTTATTGAACGTTCCCA
TTGGCG**TGCATCCAGCAGGAATTGAACCTACGAATTTGCCAATTATGAGTTGGGCGCTTTAACCATTCAGCCATG**
GATGCCTTAACAGGGATCATCGTACATCGTGAATAACCAAATTCCAATTGAAATGAAATCTTTAGGAGGAATCA

>*Disporum smilacinum*- Number of tandem repeats: 0
AAATCCATTTTCTTCCCTATGAGTTCCAGTATCGATAAGAATTCTAGTTCTTACTGTTCATATGTTATGGTATGAATATA
CCATACCAATTCGTTATGTATGGATGATGAGATTCCATTGATACAGAGCCAATTCCAATAGACTTATTGAACGTTCCCA
TTGGCG**TGCATCCAGCAGGAATTGAACCTACGAATTTGCCAATTATGAGTTGGGCGCTTTAACCATTCAGCCATG**
GATGCCTTAACAGGGATCATCGTACATCGCGAATAACCAAATTCCAATTGAAATGAAATCTTTAGGAGGAATCA

>*Alstroemeria aurea*- Number of tandem repeats: 0

AAATCCGTTTTCTTCCCTATGAGTTCCAGTATCGATAAGAATTCTAGTTCTTACTGTTCATATGTTATGGTATGAATATA
CCATACCAATTCGCTATGTATGGATGATGAGATTCCATTGATACAGAGCCAATTCCAATAGACTTATTGAACGTGCCCA
TTGGCG**TGCATCCAGCAGGAATTGAACCTACGAATTTACCAATTATGAGTTGGGCGCTTTAACCATTCAGCCATG**
GATGCTTAACAGGGATCATCGTACATCGTGAATAACCAAATTCCAATTGAAATGAAATCTTTAGGAGGAATCA

>*Bomarea edulis*- Number of tandem repeats: 0

AAATCCATTTTCTTCCCTATGAGTTCCAGTATCGATAAGAATTCTAGTTCTTACTGTTCATATGTTATGGTATGAATATA
CCATACCAATTCGTTATGTATGGATGATGAGATTCCATTGATACAGAGCCAATTCCAATAGACTTATTGAACGTTCCCA
TTGGCG**TGCATCCAGCAGGAATTGAACCTACGAATTTGCCAATTATGAGTTGGGCGCTTTAACCATTCAGCCATG**
GATGCTTAACAGGGATCATCGTACATCGTGAATAACCAAATTCCAATTGAAATGAAATCCTTAGGAGGAATCA

>*Luzuriaga radicans*- Number of tandem repeats: 0

AAATCCATTTTCTTCCCTATGAGTTCCAGTATCGATAAGAATTCTAGTTCTTACTGTTCATATGTTATGGTATGAATATA
CCATACCAATTCGTTATGTATGGATGATGAGATTCCATTGATACAGAGCCAATTCCAATAGACTTATTGAACGTTCCCA
TTGGCG**TGCATCCAGCAGGAATTGAACCTACGAATTTGCCAATTATGAGTTGGGCGCTTTAACCATTCAGCCATG**
GATGCTTAACAGGGATCATCGTACATCGTGAATAACCAAATTCCAATTGAAATGAAATCTTTAGGAGGAATCA

>*Petermannia cirrosa*- Number of tandem repeats: 0

AAATCCATTTTCTTCCCTATGAGTTCCAGTATCGATAAGAATTCTAGTTCTTACTGTTCATATGTTATGGTATGAATATA
CCATAACAATTCGTTATGTATGGATGATGAGATTCCATTGATACAGAGCCAATTCCAATAGACTTATTGAACGTTCCCA
TTGGCG**TGCATCCAGCAGGAATTGAACCTACGAATTTGCCAATTATGAGTTGGGCGCTTTAACCATTCAGCCATG**
GATGCTTAACAGGGATCATCGTACATCGTGAATAACCAAATTCCAATTGAAATGAAATCTTTAGGAGGAATCA

>*Campynema lineare*- Number of tandem repeats: 0

AAATCCATTTTCTTCCCTATGAGTTCCAGTATTGATAAGAATTCTAGTTCTTACTGTTCATATGTTATGGTATGAATATAC
CATACCAATTCGTTATATAATATATGGATGATGAGATTCCATTGATACAGAGCCAATTCCAATAGACTTATTGAACGTT
CCCATTGGCG**TGCATCCAGCAGGAATTGAACCTACGAATTTGCCAATTATGAGTTGGGCGCTTTAACCATTCAGC**
CATGGATGCTTAACAGGGATCATGGTATATCGTAAATAACCAAATTCCAATTGAAATGAAATCTTTAGGAGGAGTCA
